# Triggering Oxygen Redox Cycles in Nickel Ferrite by Octahedral Geometry Engineering for Enhancing Oxygen Evolution

**DOI:** 10.1002/advs.202409024

**Published:** 2024-12-16

**Authors:** Yang Peng, Xu Zhao, Yiqun Shao, Xin Yue, Zhuofeng Hu, Shaoming Huang

**Affiliations:** ^1^ Guangzhou Key Laboratory of Low‐Dimensional Materials and Energy Storage Devices Collaborative Innovation Center of Advanced Energy Materials School of Materials and Energy Guangdong University of Technology Guangzhou 510006 China; ^2^ School of Environmental Science and Engineering Guangdong Provincial Key Laboratory of Environmental Pollution Control and Remediation Technology Sun Yat‐sen University Guangzhou 510006 China; ^3^ School of Chemistry and Materials Science Hangzhou Institute for Advanced Study University of Chinese Academy of Sciences Hangzhou 310024 China

**Keywords:** adsorbate evolution mechanism, lattice‐oxygen‐mediated mechanism, octahedral geometrical defects, oxygen evolution reaction, spinel‐type Ni─Fe oxides

## Abstract

Spinel‐type nickel ferrite (Ni_x_Fe_3‐x_O_4_, x≤1) is a widely used electrocatalyst for the oxygen evolution reaction (OER). Due to the lower hybridization of metal‐*d* and oxygen‐*p* orbitals, the OER process on Ni_x_Fe_3‐x_O_4_ follows the sluggish adsorbate evolution mechanism (AEM). Generally, activating the lattice oxygen to trigger the lattice‐oxygen‐mediated mechanism (LOM) can enhance the OER activity. Herein, to trigger the LOM pathway while maintaining high stability, iron foam (IF)‐supported Ni_0.75_Fe_2.25_O_4_ (NiFeO) with geometrical defects of [NiO_6_] (nickel cation coordinated with six oxygen anions) units and higher ratio of Fe to Ni cations in octahedral sites (*d*‐NiFe^HR^O/IF) is prepared by ion‐exchanging with polar aprotic solvent followed by annealing. As a result, as‐synthesized *d*‐NiFe^HR^O/IF exhibits excellent activity (at 295 mV overpotential to achieve 100 mA cm^−2^), fast kinetics (Tafel slope of only 34.6 mV dec^−1^), and high stability (maintaining a current density of 100 mA cm^−2^ over 130 h) for the OER. The theoretical calculations reveal that the construction of octahedral defect in Ni_x_Fe_3‐x_O_4_ enhances the overlap of Fe‐*d* and O‐*p* orbitals, which can activate the lattice oxygen. Therefore, increasing the ratio of Fe to Ni will further improve the lattice oxygen redox activity, and thus trigger the fast LOM pathway, ultimately facilitating the OER process.

## Graphical abstract

Iron foam (IF) supported Ni_0.75_Fe_2.25_O_4_ with geometrical defects of [NiO_6_] units and higher ratio of Fe to Ni in octahedral sites (*d*‐NiFe^HR^O/IF) is synthesized. It is found defects are constructed by coordination ion exchange with polar aprotic solvent, and the annealing allows cations to refilled into defects and optimizes ratio of Fe to Ni in octahedral sites.

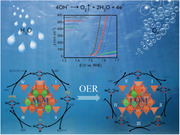

## 1 Introduction

The oxygen evolution reaction (OER) is regarded as a crucial half‐reaction of many energy conversion processes,^[^ ^1^ ^]^ e.g., electrochemical water splitting,^[^ ^2^ ^]^ metal‐air batteries,^[^ ^3^ ^]^ nitrogen reduction reaction,^[^ ^4^ ^]^ etc. However, owing to the multi‐proton‐coupled‐electron transfer processes, the OER presents intrinsic sluggish kinetics and therefore requires a considerable overpotential to overcome the energy barrier.^[^ ^5^ ^]^ To date, IrO_2_ and RuO_2_ are recognized to be state‐of‐the‐art electrocatalysts for the OER, but the scarcity and high cost hamper their widespread applications.^[^ ^6^ ^]^ Consequently, it is a key issue to design and develop low‐cost, highly active, and stable electrocatalysts for the OER.^[^ ^7^ ^]^

Due to the outstanding activity and stability, spinel oxides (AB_2_O_4_) have been deemed to be one of the most potential non‐precious metal electrocatalysts for the OER, notably nickel ferrite (Ni_x_Fe_3‐x_O_4_, x≤1).^[^ ^8^ ^]^ The crystal structure of AB_2_O_4_ consists of a cubic lattice formed by closely packed oxygen anions, with cations filling the interstices defined by the oxygen anions. Among them, transition metal (TM) cations are occupied in the octahedral (TM_oct_) and tetrahedral (TM_td_) interstices, which consist of six and four oxygen anions, respectively.^[^ ^9^ ^]^ Generally, the TM_oct_ is believed to be the real active species during the OER process because they tend to be preferentially exposed on the near surface.^[^ ^10^ ^]^ Since the oxygen anion is shared by one TM_td_ and three TM_oct_, the electronic polarization from anions to cations is weakened by the interaction between closest TM‐cations.^[^ ^3b^ ^]^ This will lower the hybridization of the metal‐*d* and oxygen‐p orbitals.^[^ ^11^ ^]^ Thus, the OER process on AB_2_O_4_ normally follows a sluggish adsorbate evolution mechanism (AEM) pathway, which involves four linearly concerned proton‐coupled‐electron steps.^[^ ^7^, ^12^ ^]^ It is greatly limited by the scaling relationship between the adsorbed energies of *OH and *OOH, which therefore results in a large theoretical overpotential of ≈370 mV.^[^ ^13^ ^]^ Recently, some electrocatalysts have reportedly exhibited enhanced OER activity to surpass the limit owing to that the OER process is via a kinetically favorable lattice‐oxygen‐mediated mechanism (LOM) pathway.^[^ ^14^ ^]^ This pathway allows the combination of lattice oxygen with oxygen radical to directly form O_2_ molecules, thereby preventing the generation of *OOH.^[^ ^15^ ^]^

Activation of lattice oxygen to improve oxygen redox cycles is a prerequisite for triggering the LOM pathway on Ni_x_Fe_3‐x_O_4_, which requires upshifting the oxygen‐*p* band or downshifting the metal‐*d* band to increase the overlap between *d*‐*p* orbitals.^[^ ^16^ ^]^ Defect engineering has become a feasible approach since creating anion or cationic vacancies has been extensively proven to efficiently tune oxygen‐*p* or metal‐*d* orbitals, respectively.^[^ ^17^ ^]^ However, constructing a single type of defect into Ni_x_Fe_3‐x_O_4_ still fails to meet industrial requirements, owing to insufficient oxygen redox activity.^[^ ^18^ ^]^ Manufacturing cationic and anionic multiple vacancies is thus believed to be able to maximize hybridization to yield satisfactory OER activity.^[^ ^12^, ^17^ ^]^ Nevertheless, controllable building cationic and anionic multiple vacancies into Ni_x_Fe_3‐x_O_4_ is more difficult than a single type of defect because of the much higher formation energy.^[^ ^19^ ^]^ Recently, limited reports have demonstrated that cationic and anionic multiple vacancies could be created into Ni─Fe layered double hydroxides (Ni─Fe LDHs) by coordinating with suitable polar aprotic solvents to dissolve cations and oxygen‐containing anions.^[^ ^20^ ^]^ This may provide an opportunity to construct multiple vacancies into Ni_x_Fe_3‐x_O_4_. Nonetheless, applying defect engineering to develop OER electrocatalysts for practical deployment still remains challenging.^[^ ^21^ ^]^ It is because the dynamic formation and migration of lattice oxygen vacancies can generate uncoordinated cations, leading to the dissolution and leaching of metal ions from the bulk phase, which severely reduces the stability of the catalyst.^[^ ^22^ ^]^ Notably, under OER conditions, the dissolution rate of Fe is significantly higher than Ni.^[^ ^22^ ^]^ Therefore, Fe leakage is a crucial challenge hindering the practical application of Ni─Fe oxides.^[^ ^20^, ^21^ ^]^ Fortunately, it has been recently suggested to compensate for Fe ions to suppress their rapid dissolution, which even achieves equilibrium between dissolution/redeposition, ultimately resulting in long‐term stability.^[^ ^23^ ^]^

Therefore, herein, to trigger the kinetically favorable LOM pathway to yield satisfactory activity while maintaining high stability, we fabricate iron foam (IF) supported Ni_0.75_Fe_2.25_O_4_ (NiFeO) with octahedral geometrical defects (*d*‐) of [NiO_6_] (nickel cation coordinated with six oxygen anions) units and higher ratio (HR) of Fe to Ni in octahedral sites (*d*‐NiFe^HR^O/IF) by ion‐exchanging with polar aprotic solvent (i.e., N, N‐Dimethylformamide, DMF) followed by annealing. Owing to that [NiO_6_] units are dissolved out from NiFeO as well as foreign Fe cations from IF are partially refilled into geometrical defects by annealing, the ratio of Fe to Ni is increased. Due to triggering the fast LOM, as‐prepared (*d*‐NiFe^HR^O/IF exhibits outstanding activity (with an overpotential of 295 mV to achieve 100 mA cm^−2^) and fast kinetics (with a Tafel slope of only 34.6 mV dec^−1^) toward OER. Because of the optimized ratio of Fe to Ni, *d*‐NiFe^HR^O/IF presents high stability for OER, which can maintain a current density of 100 mA cm^−2^ for over 130 h. The detailed mechanism of triggering the LOM pathway has been studied by the in situ characterizations and theoretical calculations.

## 2 Results and Discussion

**Figure 1:**
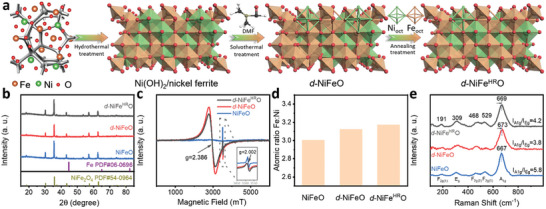
Synthesis and structural characterizations of *d*‐NiFe^HR^O/IF. a) Scheme of synthetic route for *d*‐NiFe^HR^O/IF. b) XRD patterns, c) EPR spectra, d) cationic ratio of Fe to Ni, and e) Raman spectra of *d*‐NiFe^HR^O, *d*‐NiFeO, and NiFeO, respectively.

The scheme of the synthetic route for *d*‐NiFe^HR^O/IF is shown in **Figure** **1**a, involving a hydrothermal treatment, a solvothermal treatment followed by an annealing process. The nickel nitrate hexahydrate (Ni(NO_3_)_2_·6H_2_O) and iron nitrate nine hydrate (Fe(NO_3_)_3_·9H_2_O) were used as the precursors of the Ni and Fe elements, and the IF was served as the conductive and porous substrate. In general, when the metal cations and hydroxyl anions are dissolved out from the TM‐hydroxides by coordinating and ion‐exchanging with polar aprotic solvent, e.g., DMF, the anionic and cationic defects would be created:^[^ ^20b^ ^]^

(1) $$M{\left(\mathrm{OH}\right)}_{n}+L\to M{L_{n}}^{x+}+nOH^{-}$$

where L represents the polar aprotic solvent. It is noteworthy that Ni(OH)_2_ was generated after the hydrothermal treatment as an intermediate (Figure S1, Supporting Information). Thus, the octahedral geometrical defects constructed during the solvothermal process should be mainly defects of [NiO_6_] units, and the ratio of Fe to Ni cations is increased. Subsequently, according to our previous report, foreign Fe cations from the IF substrate can be partially refilled into the octahedral defects by annealing, thereby the content of Fe cations has been further optimized.^[^ ^24^ ^]^ Additionally, IF supported Ni_0.75_Fe_2.25_O_4_ (NiFeO/IF) and IF supported Ni_0.75_Fe_2.25_O_4_ with octahedral geometrical defects (*d*‐NiFeO) were prepared through similar methods as comparisons (for details see Experimental Section).

The X‐ray diffraction (XRD) patterns of *d*‐NiFe^HR^O, *d*‐NiFeO, and NiFeO are presented in Figure 1b. The peaks of *d*‐NiFe^HR^O, *d*‐NiFeO, and NiFeO at 18.4°, 30.3°, 35.7°, 43.4°, 47.5°, 53.8°, 57.4°, and 63.0° correspond to the (111), (220), (311), (222), (400), (422), and (440) facets of the spinel NiFe_2_O_4_ (PDF#54‐0964) with the space group of *Fd*‐*3* *m* (227), representing the successful synthesis of spinel phase of nickel ferrite. The electron paramagnetic resonance (EPR) spectra of *d*‐NiFe^HR^O, *d*‐NiFeO, and NiFeO are displayed in Figure 1c. Two signals at g = 2.002 and 2.386 can be observed on the spectra of *d*‐NiFe^HR^O and *d*‐NiFeO corresponding to the existence of ionized oxygen vacancies and octahedral geometrical defects of TMO_6_ units, respectively.^[^ ^18^, ^25^ ^]^ This indicates that the octahedral geometrical defects can be successfully constructed by ion‐exchanging with DMF to dissolve out the cations and oxygen‐containing anions. Additionally, the EPR signal of *d*‐NiFeO at g = 2.386 is more intensified compared to that of *d*‐NiFe^HR^O, which indicates the higher concentration of octahedral geometrical defects in *d*‐NiFeO lattice. This implies that the octahedral geometrical defects of *d*‐NiFe^HR^O are partially refilled. Furthermore, the cationic ratios of Fe to Ni in *d*‐NiFe^HR^O, *d*‐NiFeO, and NiFeO were quantitatively analyzed by inductively coupled plasma‐atomic emission spectroscopy (ICP‐AES) (Figure 1d; Table S1, Supporting Information). The ratio of Fe to Ni in NiFeO is determined to be 3.01, and the formula of NiFeO is therefore calculated to be Ni_0.75_Fe_2.25_O_4_ The ratio in *d*‐NiFeO is obtained to be 3.12, suggesting that the cations dissolved out from NiFeO during the solvothermal process are mainly Ni. Furthermore, the ratio in *d*‐NiFe^HR^O is 3.17, confirming the partial filling of foreign Fe cations into the octahedral defects.

Raman spectra of *d*‐NiFe^HR^O, *d*‐NiFeO, and NiFeO present five Raman active modes of spinel‐type oxide, respectively (Figure 1e). On the spectra of *d*‐NiFe^HR^O, five characteristic vibrational bands (E_g_+A_1g_+3F_2_ _g_) are located at 191, 309, 468, 529, and 669 cm^−1^, respectively.^[^ ^26^ ^]^ Among them, the E_g_ and A_1_ _g_ modes at 309 and 669 cm^−1^ are related to the vibrations of tetrahedral and octahedral sub‐lattices. Given the tetrahedral geometry of the spinel oxide, the intensity ratio of A_1_ _g_ to E_g_ (I_A1g_/I_Eg_) represents the occupying degree of the octahedral interstices.^[^ ^8^, ^18^ ^]^ As a result, NiFeO exhibits an I_A1g_/I_Eg_ value of 5.8, while the ratio in *d*‐NiFeO decreases to 3.8, meaning the successful construction of the octahedral geometrical defects. Moreover, the I_A1g_/I_Eg_ value in *d*‐NiFe^HR^O is found to increase to 4.2, implying the octahedral geometrical defects are partially refilled. On the other hand, when compared to NiFeO (667 cm^−1^), the A_1_ _g_ modes of *d*‐NiFe^HR^O (669 cm^−1^) and *d*‐NiFeO (673 cm^−1^) can be observed to blue‐shift to the higher frequencies, suggesting the variations of dominant elements in the octahedral sites.^[^ ^27^ ^]^ Usually, as an inverse spinel structure, Ni cations in Ni_x_Fe_3‐x_O_4_ (x≤1) will be fully filled into octahedral interstices, while the remaining octahedral sites and all tetrahedral sites will be occupied by Fe cations.^[^ ^8b^ ^]^ In this report, the ratios of Fe_oct_ to Ni_oct_ in the octahedral sites of *d*‐NiFeO and *d*‐NiFe^HR^O are found to increase due to the dissolution of [NiO_6_] units and partial filling of foreign Fe into octahedral defects. Thus, the A_1_ _g_ modes in *d*‐NiFeO and *d*‐NiFe^HR^O blue‐shift to the higher frequencies. Similar results have been found in the Fourier transform infrared (FTIR) spectroscopy as well (Figure S2, Supporting Information).

**Figure 2:**
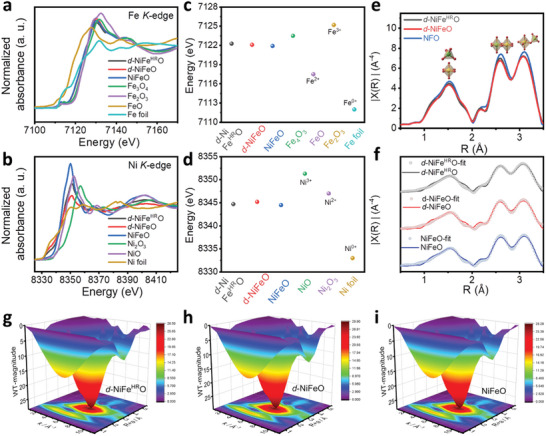
Investigations of valence states and local coordination environments. a) Fe K‐edge and b) Ni K‐edge normalized XANES spectra of *d*‐NiFe^HR^O, *d*‐NiFeO, NiFeO, and comparisons. Relationships between the valence states and the absorption energies in the first derivative of XANES spectra of *d*‐NiFe^HR^O, *d*‐NiFeO, NiFeO, and reference materials recorded at c) Fe K‐edge and d) Ni K‐edge. e) Resulting Fourier‐transform (FT) *k^3^* weighted χ(k)‐function of Fe K‐edge EXAFS spectra for *d*‐NiFe^HR^O, *d*‐NiFeO, and NiFeO. f) Experimental data and fits of the Fe K‐edge EXAFS spectra for *d*‐NiFe^HR^O, *d*‐NiFeO, and NiFeO. g–i) WT for the *k^3^* weighted EXAFS contour plots of *d*‐NiFe^HR^O, *d*‐NiFeO, and NiFeO.

The local valance states and coordination environments of *d*‐NiFe^HR^O, *d*‐NiFeO, and NiFeO were investigated by X‐ray absorption fine structure spectroscopy (XAFS). The X‐ray absorption near edge structure (XANES) spectra recorded at Fe and Ni K‐edges of *d*‐NiFe^HR^O, *d*‐NiFeO, NiFeO, and other compounds are illustrated in **Figure** **2**a,b. The valence states of Fe and Ni in these materials were studied from the highest absorption energy positions of the first derivative for XANES spectra at Fe and Ni K‐edges (Figure S3, Supporting Information). As displayed in Figure 2c,d, NiFeO presents the lowest average valence stats of Fe and Ni, while the valence states of Fe and Ni in *d*‐NiFeO both increase, owing to the construction of geometrical defects. However, after partially filling foreign Fe cations into octahedral defects, the average valence state of Fe in *d*‐NiFe^HR^O further increases, but the valence state of Ni cations decreases. This may mean that the Fe‐*d* orbitals have stronger hybridization with the O‐*p* orbitals as compared to that of Ni. Similar situations regarding the valence states have been also found in the X‐ray photoelectron spectroscopy (XPS) characterizations (Figures S4–S7, Supporting Information).

As exhibited in the Fourier transform (FT) Fe K‐edge extended X‐ray absorption fine structure (EXAFS) spectra of *d*‐NiFe^HR^O, *d*‐NiFeO, and NiFeO in Figure 2e, the peaks at around 1.5 Å belong to the metal‐oxygen (TM_oct_‐O/TM_td_‐O) bonds; the peaks at about 2.5 Å are responsible for the bonds between the closest octahedral cations (TM_oct_‐TM_oct_); and the peaks at about 3.0 Å are ascribed to the bonds between tetrahedral cations with the neighbored cations (TM_td_‐TM_oct_/TM_td_‐TM_td_).^[^ ^28^ ^]^ Additionally, the experimental EXAFS spectra of *d*‐NiFe^HR^O, *d*‐NiFeO, and NiFeO were well fitted to the structure of AB_2_O_4_ (*Fd‐3* *m (227)*) (Figure 2f; Table S2, Supporting Information). As a result, the first shell of the EXAFS spectrum for NiFeO can be fitted to be the paths of Fe_td_‐O with a coordination number (CN) of 3.988 and Fe_oct_‐O with a CN of 5.835. After constructing geometrical defects, the CN of Fe_oct_‐O in *d*‐NiFeO decreases to 5.038. Due to the partial oxidation during the annealing process, the amplitude of the TM‐O characteristic peak on the spectra of *d*‐NiFe^HR^O slightly increases, which is fitted to be a path of Fe_oct_‐O with a CN of 5.378. Furthermore, as compared to that of NiFeO (5.768), the CN of Fe_oct_‐TM_oct_ in *d*‐NiFeO decreases to 5.022, indicating the successful construction of octahedral defects. Because of partially refilling foreign Fe cations into the octahedral defects, the CN of Fe_oct_‐TM_oct_ in *d*‐NiFe^HR^O rises to 5.342. Additionally, it is believed that the foreign Fe cations only partially fill into octahedral defects, rather than tetrahedral interstices, owing to the unchanged tetrahedral characteristic peaks on the spectra of *d*‐NiFe^HR^O and *d*‐NiFeO. The variations of CN in these materials can be also observed in the wavelet transforms (WT) of the *k^3^* weighted EXAFS contour plots at Fe K‐edge (Figure 2g–i).

**Figure 3:**
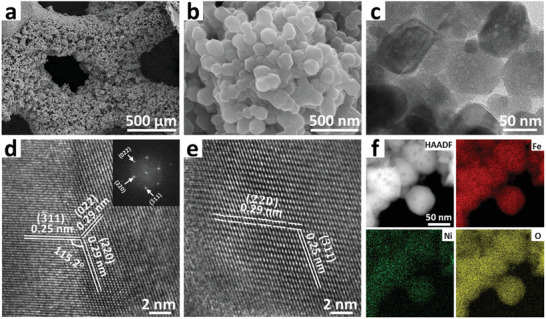
Morphological characterizations of *d*‐NiFe^HR^O/IF. a,b) SEM images of *d*‐NiFe^HR^O/IF. c–e) TEM images of *d*‐NiFe^HR^O. (f) HAADF‐STEM and corresponding elemental mapping images of *d*‐NiFe^HR^O.

The scanning electron microscopy (SEM) images of *d*‐NiFe^HR^O/IF reveal that the *d*‐NiFe^HR^O nanoplates with sizes of 50–250 nm are uniformly supported on the IF substrate (**Figure** **3**a,b; Figure S8, Supporting Information). Similar situations regarding the morphologies and sizes of *d*‐NiFeO, and NiFeO are observed as well (Figures S9 and S10, Supporting Information). As shown in the transmission electron microscopy (TEM) image, cavities with sizes ≈ 5 nm can be observed in some *d*‐NiFe^HR^O nanoplates, proving the successful dissolution of cations and anions to create octahedral geometrical defects during the solvothermal process (Figure 3c). The high‐resolution (HR)‐TEM image of *d*‐NiFe^HR^O is exhibited in Figure 3d. The lattice fringes with interplanar spacings of 0.25 and 0.29 nm are attributed to the ($\bar{3}$11) and (220) facets of the Ni_0.75_Fe_2.25_O_4_. Besides, the lattice distortions in *d*‐NiFe^HR^O can be found in the HR‐TEM image, which should be owing to the lattice strain arising from the formation of geometric defects (Figure 3e). The morphologies as well as crystal structures of *d*‐NiFeO, and NiFeO were investigated by TEM characterization as well (Figures S11 and S12, Supporting Information). Additionally, the uniform distributions of Fe, Ni, and O elements across *d*‐NiFe^HR^O can be observed on the high‐angle annular dark‐field scanning transmission electron microscopy (HAADF‐STEM) and corresponding elemental images (Figure 3f).

**Figure 4:**
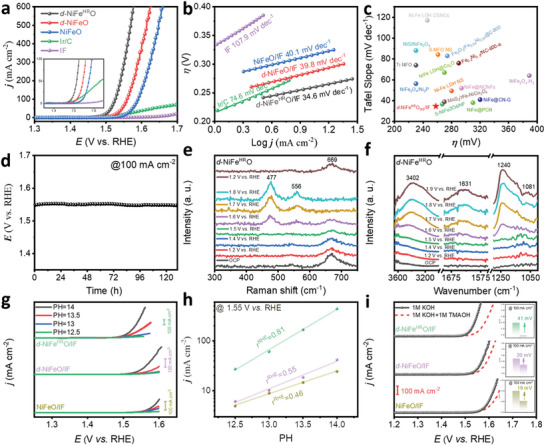
Electrocatalytic performance and mechanism of OER. a) LSV curves of OER on *d*‐NiFe^HR^O/IF, *d*‐NiFeO/IF, NiFeO/IF, Ir/C, and IF electrocatalysts in 1 M KOH at 25 °C with scan rates of 0.5 mV s^−1^. b) Tafel plots of *d*‐NiFe^HR^O/IF, *d*‐NiFeO/IF, NiFeO/IF, Ir/C, and IF electrocatalysts. c) Comparisons of OER performances in terms of kinetics and overpotential at 10 mA cm^−2^ on *d*‐NiFe^HR^O/IF and recent reported state‐of‐the‐art Ni─Fe based electrocatalysts. d) The chronoamperometric plot of OER on *d*‐NiFe^HR^O/IF electrocatalysts at 100 mA cm^−2^ in 1 M KOH with 25 °C. e) In situ electrochemical Raman spectra of *d*‐NiFe^HR^O/IF collected at various applied potentials in 1 M KOH. f) In situ FTIR spectra of *d*‐NiFe^HR^O recorded at various applied potentials in 1 M KOH. g) The polarization curves of the OER on *d*‐NiFe^HR^O/IF, *d*‐NiFeO/IF, and NiFeO/IF in KOH solutions with different pH values. h) Relationships against the OER activities on *d*‐NiFe^HR^O/IF, *d*‐NiFeO/IF, and NiFeO/IF at 1.55 V versus RHE and pH values. i) The polarization curves of OER on *d*‐NiFe^HR^O/IF, *d*‐NiFeO/IF, and NiFeO/IF in 1 M KOH and 1 M KOH + 1 M TMAOH, respectively. The inset shows the increase in overpotential of various electrocatalyst as it reaches a current density of 100 mA cm^−2^ in KOH + 1 M TMAOH than in 1 M KOH.

The OER performance of *d*‐NiFe^HR^O/IF was evaluated by a standard three‐electrode system in a 1.0 M KOH electrolyte (**Figure** **4**). For comparison, the polarization curves of OER on *d*‐NiFeO/IF, NiFeO/IF, Ir/C, and IF were tested as well. All linear sweep voltammogram (LSV) curves were *iR*‐corrected. It is worth noting that the optimized synthetic conditions have been adopted for fabricating *d*‐NiFe^HR^O/IF (Figures S13 and S14, Supporting Information). As displayed in Figure 4a, Ir/C, always used as the benchmark electrocatalyst, exhibits reasonable activity toward OER with an overpotential of 290 mV to attain 10 mA cm^−2^. The IF substrate only possesses a negligible activity, which requires a considerable overpotential of 440 mV to achieve 10 mA cm^−2^. NiFeO/IF can reach the current densities of 10 and 100 mA cm^−2^ at overpotentials of 314 and 360 mV, respectively. The activity of OER has been enhanced by constructing geometrical defects, whereas *d*‐NiFeO/IF requires overpotentials of only 285 and 328 mV to attain 10 and 100 mA cm^−2^. Furthermore, the activity of *d*‐NiFe^HR^O/IF, which can achieve the current densities at overpotentials of only 258 and 295 mV, is further increased by optimizing the ratio of Fe to Ni. Besides, a larger electrochemical surface area (ECSA) to expose more active sites is believed to contribute to the high activity of *d*‐NiFe^HR^O/IF as well (Figures S15 and S16, Supporting Information). Additionally, *d*‐NiFe^HR^O/IF presents a Tafel slope of 34.6 mV dec^−1^, which is lower than those of *d*‐NiFeO/IF (39.8 mV dec^−1^), NiFeO/IF (40.1 mV dec^−1^), Ir/C (74.6 mV dec^−1^), and IF (107.9 mV dec^−1^), confirming the fastest kinetics toward OER (Figure 4b; Figures S17 and S18, Supporting Information). Therefore, *d*‐NiFe^HR^O/IF is at a similar or even better level compared with current documented state‐of‐the‐art NiFe‐based electrocatalysts for OER in terms of the activity (overpotential to reach 10 mA cm^−2^) and kinetics (Tafel slope) (Figure 4c; Table S3, Supporting Information). Moreover, owing to the optimized ratio of Fe to Ni, *d*‐NiFe^HR^O/IF displays a high stability toward OER while maintaining the current density of 100 mA cm^−2^ for over 130 h (> 5 days) (Figure 4d). The XRD and SEM characterizations reveal that the crystal structure and morphology of *d*‐NiFe^HR^O/IF are unchanged after the stability test, proving high structural stability (Figures S19 and S20, Supporting Information).

To elucidate the catalytic mechanism of OER on *d*‐NiFe^HR^O, in situ electrochemical Raman spectroscopy was conducted in 1.0 M KOH electrolyte (Figure 4e). As a result, when increasing the potential to approach the OER region (≥1.60 V versus RHE), two distinctive bands at 477 and 556 cm^−1^ can be detected on the in situ Raman spectra of *d*‐NiFe^HR^O/IF, which are found to be potential‐dependent. These bands are related to the surface reconstruction of the electrocatalyst for generating the amorphous species (MO_x_H_y_) during the OER process.^[^ ^29^ ^]^ At the same time, due to the reconstruction, the intrinsic A_1_ _g_ bands of spinel structure are observed to gradually disappear from the in situ Raman spectra. Furthermore, the intermediates during OER on *d*‐NiFe^HR^O and NiFeO were measured by in situ electrochemical attenuated total reflection Fourier transform infrared spectroscopy (ATR‐FTIR) to investigate the catalytic mechanism (Figure 4f; Figure S21, Supporting Information). As a result, when the potentials reach the OER range (≥1.60 V vs RHE), three potential‐dependent adsorption bands concerning the intermediates generated during the AEM pathway can be detected in the in situ ATR‐FTIR spectra of *d*‐NiFe^HR^O and NiFeO, respectively. They are the *OOH at 1240 cm^−1^, the *O at 1631 cm^−1^, and the *OH at 3402 cm^−1^, respectively. Interestingly, a distinctive adsorption band at 1081 cm^−1^ can be observed in the in situ spectra of *d*‐NiFe^HR^O above the OER potentials. It is related to the *OO, which frequently represents the OO‐radical formed by combining lattice oxygen with O‐radical during the LOM pathway.^[^ ^30^ ^]^ However, this band cannot be detected in the in situ spectra of NiFeO, suggesting the LOM pathway can be successfully triggered by constructing geometrical defects and optimizing the ratio of Fe to Ni.

For investigating the kinetics of the proton‐coupled‐electron process, the OER polarization curves of *d*‐NiFe^HR^O/IF, *d*‐NiFeO/IF, and NiFeO/IF were tested at pH values of 12.5, 13.0, 13.5, and 14.0, respectively (Figure 4g). As a result, *d*‐NiFe^HR^O/IF exhibits the strongest pH‐dependent activity toward the OER than those of *d*‐NiFeO/IF and NiFeO/IF. Furthermore, the current density on the log scale as a function of pH (ρ^RHE^ = (∂(logj) / ∂pH)) is generally used as an important parameter to evaluate the degree of proton reaction on the RHE scale.^[^ ^31^ ^]^ As depicted in Figure 4h, the value of ρ^RHE^ on *d*‐NiFe^HR^O/IF is obtained to be 0.81, which is much higher than those on *d*‐NiFeO/IF (0.55) and NiFeO/IF (0.46). Frequently, the higher value of ρ^RHE^ implies the lower degree of decoupled proton‐electron transfer during the rate‐determining step (RDS) of the AEM, i.e., the deprotonation of *OOH. This therefore confirms that a higher degree of a non‐concerned proton‐electron transfer process is involved during the OER on *d*‐NiFe^HR^O/IF, namely, the LOM pathway. Additionally, unlike the AEM pathway, it will generate the O_2_2^−^ species during the LOM pathway, so the detection of the O_2_2^−^ species can verify the OER mechanism. Usually, the tetramethylammonium cation (TMA^+^) is served as a detector of the O_2_2^−^ species since it can strongly bind the O_2_2^−^ and hinder the LOM kinetics.^[^ ^32^ ^]^ As indicated in Figure 4i, the polarization curves of *d*‐NiFe^HR^O/IF, *d*‐NiFeO/IF, and NiFeO/IF were measured in the electrolyte of 1 M KOH + 1 M TMAOH. As a result, the overpotential of OER on *d*‐NiFe^HR^O/IF reaching the current density of 100 mA cm^−2^ increases by 41 mV compared to that in 1 M KOH. This is significantly higher than those of *d*‐NiFeO/IF and (20 mV) and NiFeO/IF (19 mV), indicating the stronger binding between TMA^+^ and O_2_2^−^, and proven that the OER on *d*‐NiFe^HR^O/IF undergoes the LOM pathway.

**Figure 5:**
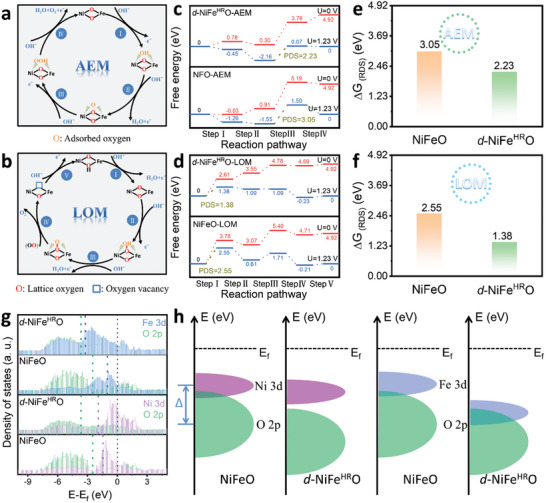
OER mechanism analysis based on DFT calculations. Schematic illustration of the OER process via a) the AEM pathway and b) the LOM pathway. OER Gibbs free energy diagrams based on *d*‐NiFe^HR^O and NiFeO through the c) AEM pathway and d) LOM pathway, respectively. Histograms of energy barriers during the PDS of the OER process on *d*‐NiFe^HR^O and NiFeO via the e) AEM pathway and f) LOM pathway (U = 1.23 V). The calculated g) PDOS and h) the schematic band diagrams of *d*‐NiFe^HR^O and NiFeO.

To clarify the origin of triggering the LOM pathway during the OER on *d*‐NiFe^HR^O density functional theory (DFT) simulations were performed to study the catalytic mechanism (**Figure** **5**). Two calculated models of Ni_0.75_Fe_2.25_O_4_ and Ni_0.75_Fe_2.25_O_4_ with [NiO_6_]‐geometrical defects were established to represent NiFeO and *d*‐NiFe^HR^O, respectively. As depicted in Figure 5a, commonly, the OER process on the surface of electrocatalysts via the AEM pathway involves the adsorption and the transformation of three key reaction intermediates, including *OH, *O, and *OOH.^[^ ^33^ ^]^ It is worth noting that the step to generate the *OOH is frequently believed to be the RDS, according to the scaling relationship. While, it is generally believed that the LOM pathway consists five basic steps with four intermediates, i.e., *O_L_, *O_L_O_a_H_a_, *O_L_O_a_, and V_O_, where O_L_ represents the lattice oxygen, O_a_ denotes as the adsorbed oxygen, O_a_H_a_ refers to the adsorbed hydroxyl species, and V_O_ means the oxygen vacancies (Figure 5b).^[^ ^34^ ^]^ As presented in Figure 5c,d, Gibbs free energy diagrams of the OER on *d*‐NiFe^HR^O and NiFeO through the AEM and LOM pathways were calculated based on the optimized structures and energies of adsorbed intermediates on the surfaces of the electrocatalysts (Figures S22–S25, Supporting Information). As a result, for the AEM pathway, *d*‐NiFe^HR^O exhibits a lower energy barrier in the potential determining‐step (PDS, *O→*OOH) of 2.23 eV as compared to NiFeO (3.05 eV) (Figure 5e). Meanwhile, as displayed in Figure 5f, the first electrochemical deprotonation step (step I) in the LOM pathway is proven to be the PDS for both *d*‐NiFe^HR^O and NiFeO, which present energy barriers of 1.38 and 2.55 eV, respectively. This indicates that the OER on the electrocatalyst exhibits higher activity through the LOM pathway compared to the AEM pathway, and *d*‐NiFe^HR^O is more active than NiFeO via both pathways. Besides, DFT calculations indicates that the surface reconstruction can further enhance the OER activity on *d*‐NiFe^HR^O (Figure S26, Supporting Information).

To understand the variation in the catalytic mechanism of OER on *d*‐NiFe^HR^O and NiFeO, the projected density of state (PDOS) and *d/p* band centers were analyzed (Figure 5g; Figure S27, Supporting Information). After constructing geometrical defects, the Fe‐*d* and Ni‐*d* band centers downshift from ‐0.98 and ‐1.43 eV to ‐3.20 and ‐1.93 eV, respectively. The downshifting *d* band centers will weaken the bonding strength of the intermediates and lower the formation energy barriers, which therefore enhances the AEM pathway. Additionally, although the O‐*p* band center decreases from ‐2.46 to ‐3.65 eV, the difference between Fe‐*d* and O‐*p* band centers (Δ_Fe/O_) is reduced from 2.48 to 0.45 eV after building defects (Figure 5h). This indicates that the hybridization of Fe‐*d* and O*‐p* orbitals is extensively enhanced and the covalency of the Fe‐O bond is thus strengthened. However, the Δ_Ni/O_ increases from 1.03 to 1.72 eV, indicating the decreased overlap between Ni*‐d* and O‐*p* bands. This shift brings the O *2p* states closer to the Fermi level, where they exhibit partially filled states. This electronic structure adjustment suggests that the presence of defects promotes the formation of nonbonding O *2p* states near the Fermi level, thereby enhancing the reactivity of lattice oxygen. In orbital theory terms, these nonbonding O orbitals now have an increased likelihood of participating in electron transfer processes, as their energy aligns closer to the Fermi level, reducing the energy barrier for redox reactions. Thus, *d*‐NiFe^HR^O enables lattice oxygen activation by shifting the electronic structure to favor unoccupied or partially occupied O *2p* states that are conducive to catalytic reactions.^[^ ^35^ ^]^ Additionally, the observed downshift of Fe *3d* states in *d*‐NiFe^HR^O can result in more localized nonbonding O orbitals, as the decreased overlap reduces the strength of bonding interactions. Accordingly, constructing octahedral geometrical defects of [NiO_6_] units in Ni_0.75_Fe_2.25_O_4_ significantly optimizes the adsorption of intermediates during the AEM pathway for enhancing the OER activity. Meanwhile, it can greatly enhance the hybridization of Fe‐*d* and O‐*p* orbitals for activation of the lattice oxygen neighbored Fe cations. Thus, increasing the cationic ratio of Fe to Ni by annealing to partially fill foreign Fe into the defects further greatly boosts the lattice oxygen redox activity, which triggers the LOM pathway and ultimately facilitates the OER process.

## 3 Discussion

In summary, to trigger the kinetically favorable LOM pathway to yield satisfactory activity while maintaining high stability, IF supported Ni_0.75_Fe_2.25_O_4_ with geometrical defects of [NiO_6_] units and higher ratio of Fe to Ni in octahedral sites (*d*‐NiFe^HR^O/IF) was prepared. The octahedral geometrical defects were built by coordinating and ion‐exchanging with DMF to dissolve out the [NiO_6_] units from NiFeO. The ratio of Fe to Ni was increased by etching out [NiO_6_] units followed by annealing to partially refill foreign Fe cations from the IF into the octahedral defects. The EPR and Raman spectroscopies confirmed the successful construction and partial filling of the octahedral geometrical defects in NiFeO. The ICP characterization proved that the ratios of Fe to Ni were increased after constructing defects and annealing, respectively. The cavity and lattice distortion arising from building geometrical defects were observed through TEM characterization. The synchrotron radiation measurements revealed the variations of valences states and coordination environments, which demonstrated the construction of geometrical defects as well as partially filling Fe cations into the defects. As a result, as‐fabricated *d*‐NiFe^HR^O/IF exhibited efficient activity with an overpotential of 295 mV to reach 100 mA cm^−2^. Meanwhile, it presented a Tafel slope of 34.6 mV dec^−1^, indicating a fast kinetics for the OER. Owing to the optimized content of Fe cations, *d*‐NiFe^HR^O/IF possessed high stability toward the OER while maintaining the current density of 100 mA cm^−2^ for over 48 h. In situ spectroscopies and other electrochemical measurements revealed that the OER process on *d*‐NiFe^HR^O/IF was via the LOM pathway. Furthermore, according to the detailed DFT calculations, the construction of octahedral defect in NiFeO greatly enhanced the overlap of Fe‐*d* and O‐*p* orbitals, which can promote the AEM pathway together with activating the lattice oxygen. Subsequently, increasing the ratio of Fe to Ni in NiFeO would further improve the lattice oxygen redox activity, and thus trigger the fast LOM pathway, ultimately facilitating the OER process. This study is of significance for designing the developing highly efficient and durable Ni─Fe oxides based electrocatalysts for the OER.

## 4 Experiment Section

### Synthesis—Chemicals

Ferric nitrate nine‐hydrate (Fe(NO_3_)_3_·9H_2_O, AR), Sodium dodecyl sulfate (SDS, C_16_H_33_NaO_4_S, AR), and potassium hydroxide (KOH, AR) were purchased from Shanghai Macklin Biochemical Technology Co., Ltd. Nickel nitrate hexahydrate (Ni(NO_3_)_2_·6H_2_O, AR), and urea (CO(NH_2_)_2_, AR) were purchased Shanghai Aladdin Biochemical Technology Co., Ltd. N, N‐Dimethylformamide (DMF, C_3_H_7_NO, AR), acetone and anhydrous ethanol were purchased from Tianjin Damao Chemical Reagents Factory. Hydrochloric acid (HCl, AR) was purchased from Chengdu Cologne Chemical Co. Iron Foams (IF) was purchased from Kunshan Jiayisheng Electronics Co., Ltd. Deionized water was homemade.

Synthesis of IF supported Ni_0.75_Fe_2.25_O_4_ with geometrical defects (d‐) of [NiO_6_] units and higher ratio (HR) of Fe to Ni in octahedral sites (d‐NiFe^HR^O/IF): First, a piece of IF (20 mm × 20 mm × 1.5 mm) was ultrasonically treated with 50 ml of 1 M HCl for 30 min to remove the surface oxides. Then, the resulting IF substrate was washed several times with deionized water. Afterward, 60 ml of deionized water was put into a Teflon‐lined autoclave (100 ml). Subsequently, 0.2 mM of Ni(NO_3_)_2_•6H_2_O, 0.4 mM of Fe(NO_3_)_3_•9H_2_O, 0.4 mM of SDS, and 4 mM of CO(NH_2_)_2_ were put into the autoclave and then ultrasonically treated for 30 min. The IF was put into the resulting solution, and then the autoclave was transformed to an electronic oven and heat‐treated at 120 °C for 10 h. After cooling down to room temperature, the obtained complex was washed three times with deionized water and ethanol. 20 ml of DMF, 2.5 ml of ethanol, 2.5 ml of deionized water, and the resulting complex were then put into a Teflon‐lined autoclave (50 ml). The autoclave was transformed to an electronic oven and heat‐treated at 150 °C for 30 h. After cooling down to room temperature, the obtained complex was washed with acetone, ethanol and deionized water. Finally, it was dried in a vacuum oven at 60 °C for 8 h and then annealed under a nitrogen atmosphere at 400 °C for 2 h.

### Synthesis of IF Supported Ni_0.75_Fe_2.25_O_4_ with Geometrical Defects (d‐) of [NiO_6_] Units (d‐NiFeO/IF)

The annealing process was not carried out and the other synthesis processes were as same as that for *d*‐NiFe^HR^O/IF.

### Synthesis of IF Supported Ni_0.75_Fe_2.25_O_4_ (NiFeO/IF)

The solvothermal treatment was not carried out and the other synthesis processes were as same as that for *d*‐NiFe^HR^O/IF.

## Conflict of Interest

The authors declare no conflict of interest.

## Supporting information



Supporting Information

## Data Availability

Research data are not shared.

## References

[1] a) L. Chong , G. Gao , J. Wen , H. Li , H. Xu , Z. Green , J. D. Sugar , A. J. Kropf , W. Xu , X. M. Lin , H. Xu , L. W. Wang , D. J. Liu , Science 2023, 380, 609; 37167381 10.1126/science.ade1499

[2] a) Y. Wang , Y. Jiao , H. Yan , G. Yang , C. Tian , A. Wu , Y. Liu , H. Fu , Angew. Chem. Int. Ed. 2022, 61, e202116233; 10.1002/anie.202116233 34984764

[3] a) R. Li , W. Fan , P. Rao , J. Luo , J. Li , P. Deng , D. Wu , W. Huang , C. Jia , Z. Liu , Z. Miao , X. Tian , ACS Nano 2023, 17, 18128; 37690054 10.1021/acsnano.3c04945

[4] Y. Hong , Q. Wang , Z. Kan , Y. Zhang , J. Guo , S. Li , S. Liu , B. Li , Chin. J. Catal. 2023, 52, 50.

[5] a) S. Luo , C. Dai , Y. Ye , Q. Wu , J. Wang , X. Li , S. Xi , Z. J. Xu , Angew. Chem. Int. Ed. 2024, 63, e202402184; 10.1002/anie.202402184 38750660

[6] a) T. Wu , S. Sun , J. Song , S. Xi , Y. Du , B. Chen , W. A. Sasangka , H. Liao , C. L. Gan , G. G. Scherer , L. Zeng , H. Wang , H. Li , A. Grimaud , Z. J. Xu , Nat. Catal. 2019, 2, 763;

[7] a) Y. Yan , J. Lin , K. Huang , X. Zheng , L. Qiao , S. Liu , J. Cao , S. C. Jun , Y. Yamauchi , J. Qi , J. Am. Chem. Soc. 2023, 145, 24218; 37874900 10.1021/jacs.3c08598

[8] a) X. Yan , W.‐D. Zhang , H. Xu , B. Liu , M. Hu , J. Liu , Z.‐G. Gu , J. Colloid Interface Sci. 2023, 632, 44; 36403376 10.1016/j.jcis.2022.11.054

[9] Y. Zhou , S. Sun , C. Wei , Y. Sun , P. Xi , Z. Feng , Z. J. Xu , Adv. Mater. 2019, 31, 1902509. 10.1002/adma.201902509 31361056

[10] C. Wei , Z. Feng , G. G. Scherer , J. Barber , Y. Shao‐Horn , Z. J. Xu , Adv. Mater. 2017, 29, 1606800. 10.1002/adma.201606800 28394440

[11] a) R. Chen , Z. Wang , S. Chen , W. Wu , Y. Zhu , J. Zhong , N. Cheng , ACS Energy Lett. 2023, 8, 3504;

[12] J. Sun , H. Xue , Y. Zhang , X. L. Zhang , N. Guo , T. Song , H. Dong , Y. Kong , J. Zhang , Q. Wang , Nano Lett. 2022, 22, 3503. 35315671 10.1021/acs.nanolett.1c04425

[13] a) Z.‐F. Huang , J. Song , Y. Du , S. Xi , S. Dou , J. M. V. Nsanzimana , C. Wang , Z. J. Xu , X. Wang , Nat. Energy 2019, 4, 329;

[14] a) C. H. Jia , X. P. Xiang , J. Zhang , Z. Y. He , Z. H. Gong , H. J. Chen , N. Zhang , X. W. Wang , S. J. Zhao , Y. Chen , Adv. Funct. Mater. 2023, 33, 2301981;

[15] N. Zhang , Chai, Y. , Energy Environ. Sci. 2021, 14, 4647.

[16] S. Wang , X. Liu , X. Chen , K. Dastafkan , Z.‐H. Fu , X. Tan , Q. Zhang , C. Zhao , J. Energy Chem. 2023, 78, 21.

[17] a) Y. H. Wang , L. Li , J. Shi , M. Y. Xie , J. Nie , G. F. Huang , B. Li , W. Hu , A. Pan , W. Q. Huang , Adv. Sci. 2023, 10, 2303321; 10.1002/advs.202303321 PMC10646268 37814357

[18] Y. Peng , C. R. Huang , J. L. Huang , M. Feng , X. Z. Qiu , X. Yue , S. M. Huang , Adv. Funct. Mater. 2022, 32, 2201011.

[19] a) J. Zheng , X. Peng , Z. Xu , J. Gong , Z. Wang , ACS Catal. 2022, 12, 10245;

[20] a) Y. Wang , S. Tao , H. Lin , G. Wang , K. Zhao , R. Cai , K. Tao , C. Zhang , M. Sun , J. Hu , B. Huang , S. Yang , Nano Energy 2021, 81, 105606;

[21] Y. Zhai , X. Ren , Y. Sun , D. Li , B. Wang , S. Liu , Appl. Catal., B 2023, 323, 122091.

[22] F.‐Y. Chen , Z.‐Y. Wu , Z. Adler , H. Wang , Joule 2021, 5, 1704.

[23] J. Liu , W. Du , S. Guo , J. Pan , J. Hu , X. Xu , Adv. Sci. 2023, 10, 2300717. 10.1002/advs.202300717 PMC10238203 37026683

[24] X. Yue , X. Qin , Y. Chen , Y. Peng , C. Liang , M. Feng , X. Qiu , M. Shao , S. Huang , Adv. Sci. 2021, 8, 2101653. 10.1002/advs.202101653 PMC8425945 34245109

[25] Y. Li , Y. Li , X. Xu , C. Ding , N. Chen , H. Ding , A. Lu , Chem. Geol. 2019, 504, 276.

[26] M. Ya , J. Wang , G. Li , G. Gao , X. Zhao , J. Cui , H. Wu , L. Li , ACS Sustainable Chem. Eng. 2023, 11, 744.

[27] a) S. Liu , B. Zhang , Y. Cao , H. Wang , Y. Zhang , S. Zhang , Y. Li , H. Gong , S. Liu , Z. Yang , J. Sun , ACS Energy Lett. 2022, 8, 159;

[28] Y. Zhou , S. Sun , S. Xi , Y. Duan , T. Sritharan , Y. Du , Z. J. Xu , Adv. Mater. 2018, 30, 1705407. 10.1002/adma.201705407 29356120

[29] a) L. Gao , X. Cui , Z. Wang , C. D. Sewell , Z. Li , S. Liang , M. Zhang , J. Li , Y. Hu , Z. Lin , Proc. Natl. Acad. Sci. USA 2021, 118, e2023421118; 33558243 10.1073/pnas.2023421118 PMC7896297

[30] J. Zhu , S. Zi , N. Zhang , Y. Hu , L. An , P. Xi , Small 2023, 19, 2301762. 10.1002/smll.202301762 37150854

[31] a) X. Xu , Y. Pan , Y. Zhong , C. Shi , D. Guan , L. Ge , Z. Hu , Y. Y. Chin , H. J. Lin , C. T. Chen , H. Wang , S. P. Jiang , Z. Shao , Adv. Sci. 2022, 9, 2200530; 10.1002/advs.202200530 PMC9108636 35306740

[32] a) X. Chen , Q. Wang , Y. Cheng , H. Xing , J. Li , X. Zhu , L. Ma , Y. Li , D. Liu , Adv. Funct. Mater. 2022, 32, 2112674;

[33] Y. Chen , J. Xu , Y. Chen , L. Wang , S. Jiang , Z. H. Xie , T. Zhang , P. Munroe , S. Peng , Angew. Chem. Int. Ed. 2024, 63, e202405372. 10.1002/anie.202405372 38659283

[34] a) Y. Wang , X. Ge , Q. Lu , W. Bai , C. Ye , Z. Shao , Y. Bu , Nat. Commun. 2023, 14, 6968; 37907458 10.1038/s41467-023-42728-y PMC10618233

[35] X. Ren , Y. Zhai , N. Yang , B. Wang , S. (Frank) Liu , Adv. Funct. Mater. 2024, 34, 2401610.

