# Supplementary material for: Triggering Oxygen Redox Cycles in Nickel Ferrite by Octahedral Geometry Engineering for Enhancing Oxygen Evolution
Source: Adv Sci (Weinh). 2024 Dec 16;12(5):2409024. doi: 10.1002/advs.202409024 (PMC11792042; doi:10.1002/advs.202409024)
Supplement: Supplementary file 1 — Supporting Information [file ADVS-12-2409024-s001.docx]

Supporting information

**Triggering Oxygen Redox Cycles in Nickel Ferrite by Octahedral Geometry Engineering for Enhancing Oxygen Evolution**

*Yang Peng, Xu Zhao, Yiqun Shao, Xin Yue*, Zhuofeng Hu* and Shaoming Huang**

**1. Experiment Details**

**1.1. Electrochemical Measurements**

Electrochemical tests of OER were carried out using a typical three-electrode system on an Autolab PGSTAT 204 electrochemical workstation. The working electrodes were *d*-NiFe^HR^O/IF, *d*-NiFeO/IF, NiFeO/IF, and IF with sizes of 10.0 mm×10.0 mm. A homemade reversible hydrogen electrode (RHE) and a graphite rod were applied as the reference electrode and counter electrode respectively. The commercial Ir/C catalyst served as a comparison was dispersed in 2 ml mixture of 1.5 ml ethanol + 0.5 ml Nafion (0.5 wt%, DuPont, USA) mixture by ultrasonically treating to form a well-dispersed catalyst ink. The catalyst ink was dropped onto a glassy carbon electrode with a diameter of 5 mm and then dried with an infrared lamp, and the loading of Ir on the electrode was 102 μg cm^-2^. The polarization curves were tested in 1 M KOH solution at 25 ^o^C with a scan rate of 0.5 mV s^-1^, and all polarization curves were *iR*-corrected. The Electrochemical Impedance Spectroscopy (EIS) was performed in 1 M KOH for 1.45 V to 1.55 V vs. RHE. Electrochemical surface area (ECSA) was measured by cyclic voltammetry in 1.0 M KOH solution in the potential range from 1.0 V to 1.1 V vs. RHE with a scan rate from 1 to 10 mV s^-1^. The stability test was performed using chronopotentiometric measurements at 100 mA cm^-2^ for 48 h.

***In situ characterizations:*** Electrochemical *in situ* Raman spectroscopy was performed on a Renishaw plc in Via Qontor Laser Microscopic Confocal Raman Spectrometer. An electrochemical *in situ* Raman H-type spectral cell (Beijing Scistar Technology Co., Ltd.) was used for the *in situ* Raman characterizations during the OER tests. Electrochemical tests of the OER were carried out using a typical three-electrode system on an Iviumstat electrochemical workstation. A glassy carbon electrode (with an area of 0.196 cm^2^) was used as the working electrode. An Ag/AgCl and a Pt electrode were applied as the reference electrode and the counter electrode respectively. The electrolyte is 1 M KOH. Before *in situ* electrochemical Raman experiments, the electrocatalysts were electrochemically activated. The electrode potential ramped up from open circuit potential (OCP) to 1.8 V vs. RHE, and the corresponding Raman spectra were collected.

Electrochemical *in situ* attenuated total reflection Fourier transform infrared spectroscopy (ATR-FTIR) tests used a 20 mm diameter Si crystal as the ATR crystal. To detect the signals of adsorbed species, a highly sensitive mercury telluride (MCT) detector as well as a high luminous flux optical path accessory were used. Spectral signs were collected by Nicolet iS50 FTIR spectrometer. A rough Au film with good electrical conductivity was deposited on the ATR crystal to enhance the IR absorption by the local electric field enhancement effect of the surface plasma. In the three-electrode electrochemical system, a catalyst film was coated on the Si crystal covered with Au film on the surface as the working electrode, and a platinum wire and silver/silver chloride electrode were used as the counter electrode and reference electrode, respectively. The connection to the external circuit was made through a copper foil. The test procedure was carried out on an iviumstat electrochemical workstation with the electrode potential ramped up from OCP to 1.9 V vs. RHE, followed by recording of IR spectra. All spectral data were collected over a wave number range of 4000 to 650 cm^-1^ with a resolution of 4 cm^-1^ and each spectrum was the average of 32 scans.

**1.2. Materials characterizations**

X-ray diffraction (XRD) characterizations were carried out on a Rigaku SmartLab 9kw X-ray diffractometer. Scanning Electron Microscopy (SEM) measurements were taken on a ThermoFisher Apreo C Scanning Electron Microscopy. Transmission Electron Microscopy (TEM) was performed on a FEI Talos F200S 200 kV scanning/transmission electron microscope. X-ray photoelectron spectroscopy (XPS) characterizations were carried out on a Thermo Fisher Escalab 250Xi X-ray photoelectron spectroscopy. Raman spectroscopy was performed on a Renishaw plc Raman spectrometer. Electron paramagnetic resonance (EPR) measurements were performed on a Bruker A300 electron paramagnetic resonance popper.

***XAS analysis:*** X-ray absorption fine structures characterizations were performed on Fe and Ni K-edges of various composites at room temperature in the transmission mode with silicon drift fluorescence detector at beamline TLS07A1 of National Synchrotron Radiation Research Center (NSRRC) operated with a Si (111) double crystal monochromator. The synchrotron was detected at 1.5 GeV and 250 mA. *d*-NiFe^HR^O, *d*-NiFeO, and NiFeO were separated from IF by ultrasonically treating in alcohol for over 6 h before XAFS measurement. The photon energy was analyzed with the first inflection point of Fe K-edge in Fe metal foil and Ni K-edge in Ni metal foil. All acquired data were fitted and analyzed by Athena and Artemis software.^[S1]^ As known, the position of the highest peak of the first derivative for XANES spectrum at Fe K-edge on Fe foil is located at 7112.0 eV as well as 8333 eV for Ni foil in Ni K-edge. Thus, XANES spectra of *d*-NiFe^HR^O, *d*-NiFeO, NiFeO, and comparisons have been calibrated.

**1.3. DFT Calculations**

All spinel calculations were performed in the framework of the density functional theory (DFT) using the Projector Augmented Plane-Wave (PAW) method, as implemented in the Vienna ab initio simulation package.^[S2]^ The generalized gradient approximation proposed by Perdew, Burke, and Ernzerhof was selected to describe the exchange-correlation potential.^[S3]^ The long range van der Waals interaction was described by the DFT-D_3_ approach.^[S4]^ The cut-off energy of the plane wave was set to 450 eV. The energy criterion was set to 10^−5^ eV in the iterative solution of the Kohn-Sham equation. The structural model was constructed based on experimental data. A slab p (9 Å × 12 Å × 27Å) was used and the Brillouin zone integration was completed by a Gamma-centred k-point grid to simulate both materials (*d*-NiFe^HR^O, NiFeO). A vacuum layer of >15 Å was added in the vertical direction of the plate sheet to avoid artificial interactions between the periodic images. All the structures were relaxed until the residual forces on the atoms declined to less than 0.03 eV/Å. The Gibbs free energy change were obtained based on the widely accepted OER four-electron transfer mechanism based on the previous report.^[S5]^

**2. Additional Figures**

The XRD patterns of the intermediates for preparing *d*-NiFe^HR^O/IF after the hydrothermal and solvothermal treatment, respectively, are shown in Figure S1a. The results indicate that the spinel-type nickel ferrite phase has been formed after the hydrothermal treatment, and the phase is well-maintained after the construction of geometrical defects. The Raman and FTIR spectra of the intermediate after the hydrothermal treatment are presented in Figure S1b and c. As a result, two vibrational bands at 454 and 525 cm^-1^ can be detected on the Raman spectrum, which is ascribed to the characteristic E_g_ and A_1g_ modes of Ni(OH)_2_.^[S6]^ Similar results have been found in the FTIR spectrum.^[S7]^ It indicates that Ni(OH)_2_ was generated onto spinel-type nickel ferrite after the hydrothermal treatment as an intermediate.


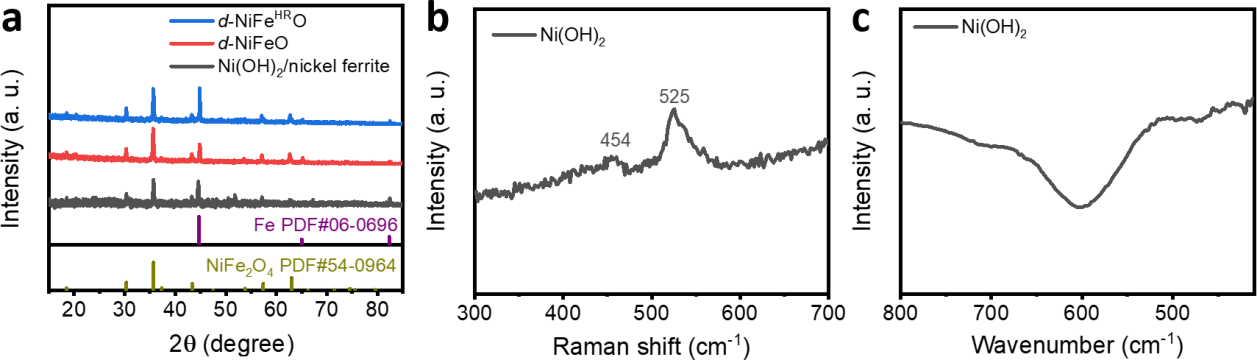


**Figure S1. (**a) The XRD patterns of *d*-NiFe^HR^O/IF and the intermediates after hydrothermal and solvothermal treatment, respectively. (b) Raman and (c) FTIR spectra of intermediates after hydrothermal process, respectively.

The FTIR spectra of *d*-NiFe^HR^O, *d*-NiFeO, and NiFeO in the range of 800 to 400 cm^-1^ are illustrated in Figure S2. The absorption bands at 457 cm^-1^ are attributed to the vibration of metal-oxygen bonding in the octahedral sites, while the bands at 580 cm^-1^ are ascribed to the vibration of metal-oxygen bonding in the tetrahedral sites.^[S8]^


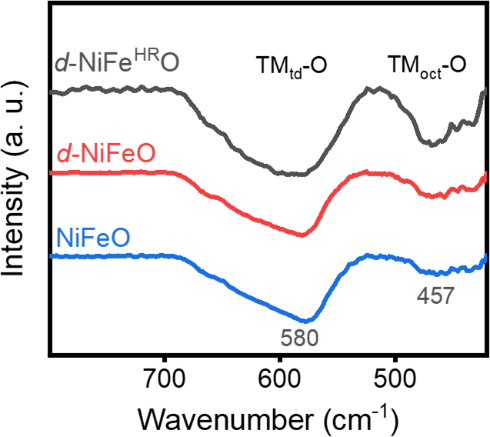


**Figure S2.** The FTIR spectra of *d*-NiFe^HR^O, *d*-NiFeO, and NiFeO.

The valence states of Fe and Ni in various materials were analyzed from the absorption energy positions of the highest peak of the first derivative for XANES spectra at Fe and Ni K-edges (Figure S3).


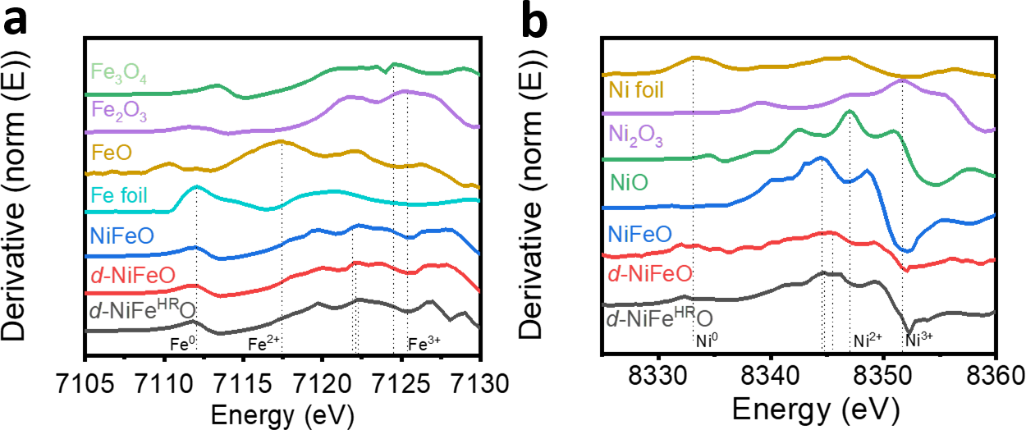


**Figure S3**. (a) The first derivative of XANES spectra at Fe K-edge of *d*-NiFe^HR^O, *d*-NiFeO, NiFeO and comparisons. (b) The first derivative of XANES spectra at Ni K-edge of *d*-NiFe^HR^O, *d*-NiFeO, NiFeO, and comparisons.


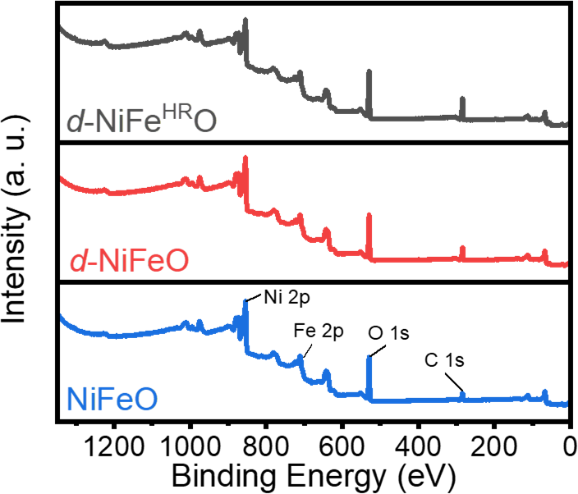


**Figure S4.** Survey XPS spectra of *d*-NiFe^HR^O/IF, *d*-NiFeO/IF, and NiFeO/IF.

The XPS spectra of *d*-NiFe^HR^O/IF, *d*-NiFeO/IF, and NiFeO/IF in Fe 2p are shown in Figure S5. As previous reports, in an inverse spinel structure, Fe^2+^ only occupies the tetrahedral sites. But Fe^3+^ occupies not only octahedral sites but also tetrahedral sites. As a result, the binding energies (BEs) of XPS spectra in Fe 2p loaded at 710.1 and 712.4 eV were attributed to Fe^2+^ and Fe^3+^, respectively. In addition, two satellite peaks at 715.6 and 719.1 eV belong to satellite peaks of Fe^2+^ and Fe^3+^, respectively.^[S9]^


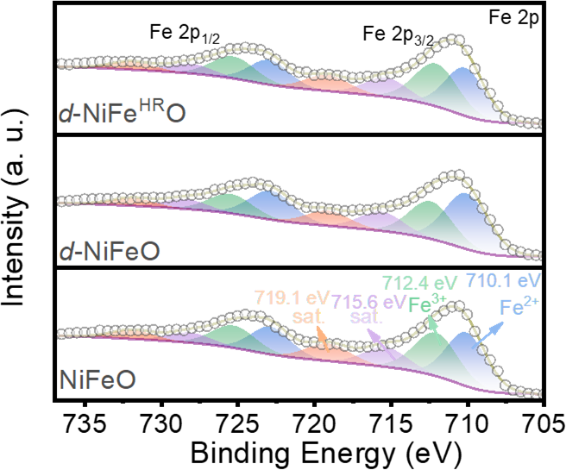


**Figure S5**. The XPS spectra of *d*-NiFe^HR^O/IF, *d*-NiFeO/IF, and NiFeO/IF in Fe 2p.

The XPS spectra in Ni 2p are depicted in Figure S6. The BEs at 854.4 and 856.1 eV are attributed to Ni^2+^ and Ni^3+^, respectively. Meanwhile, the satellite peaks at 861.2 and 864.8 eV are contributed to Ni^2+^ and Ni^3+^.^[S10]^


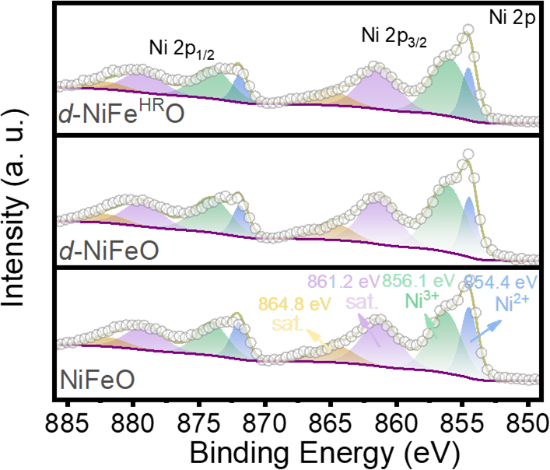


**Figure S6**. The XPS spectra of *d*-NiFe^HR^O/IF, *d*-NiFeO/IF, and NiFeO/IF in Ni 2p.

The XPS spectra in O 1s are displayed in Figure S7. The BE at 530.1 eV belongs to the TM-O bond. The BE at 531.7 eV can be attributed to the oxygen vacancy. In addition, the BE at 533.1 eV is due to the adsorption of hydroxyl groups of H_2_O molecules on the surface (Figure S7).^[S11]^


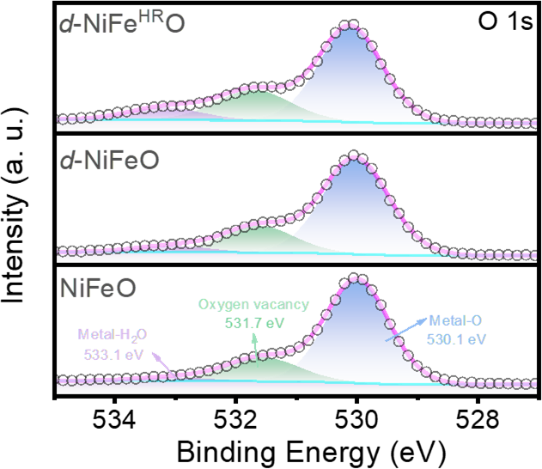


**Figure S7**. The XPS spectra of *d*-NiFe^HR^O/IF, *d*-NiFeO/IF, and NiFeO/IF in O 1s.

The SEM image of IF is shown in Figure S8. The porous and conductive IF was served as the substrate for electrocatalysts, which is beneficial for the adsorption of adsorbate.


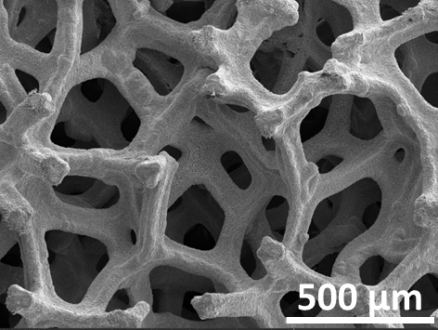


**Figure S8.** The SEM image of IF substrate.

The SEM images of *d-*NiFeO/IF are shown in Figure S9. As a result, *d-*NiFeO/IF exhibits nanoplate-like morphology with size of 100-250 nm.


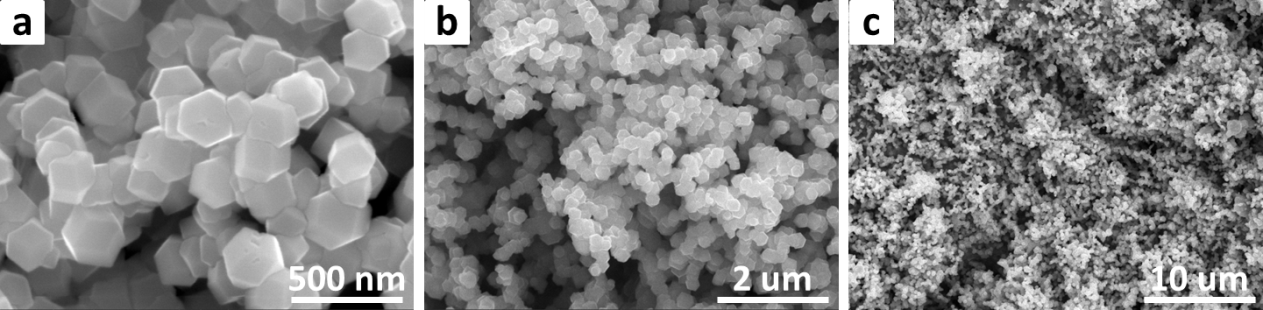


**Figure S9.** SEM images of *d-*NiFeO/IF.

The SEM images of NiFeO/IF are shown in Figure S10. As a result, NiFeO/IF presents nanoplate-like morphology with size of 50-150 nm.


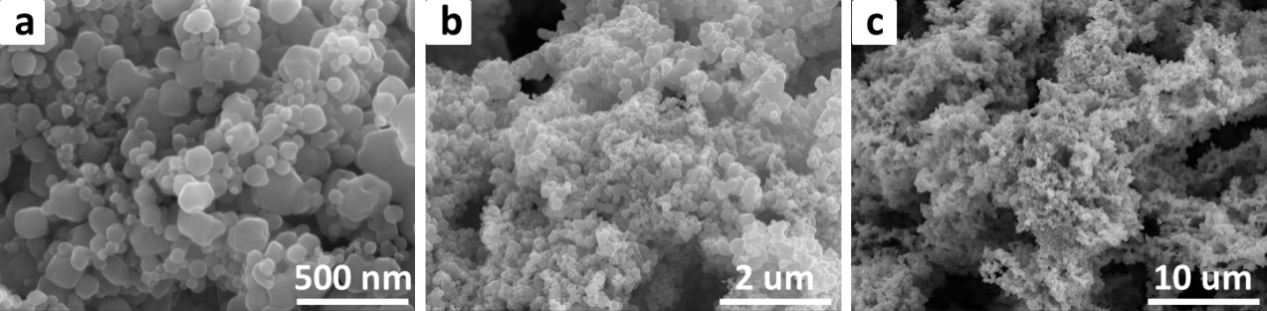


**Figure S10.** The SEM images of NiFeO/IF.

The TEM images of *d-*NiFeO are presented in Figure S11. A typical TEM image of *d-*NiFeO with sizes of 20-150 nm is shown in Figure S11a. Figure S11b shows a HR-TEM image of the edge for *d-*NiFeO. The crystalline indices of 0.30 and 0.21 nm correspond to the (220) and (311) facets of NiFe_2_O_4_, as the results of SAED in Figure S11c.


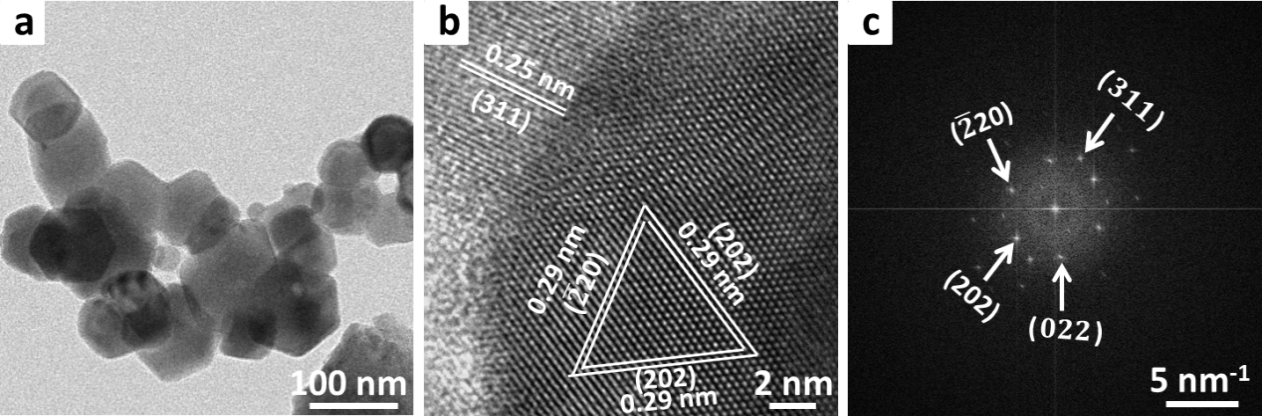


**Figure S11.** The TEM and SAED images of *d-*NiFeO.

The TEM images of NiFeO are shown in Figure S12. A typical TEM image of NiFeO with sizes of 30-250 nm is shown in Figure S12a. Figure S12b shows a HR-TEM image of the edge for NiFeO. The crystalline index of 0.25 corresponds to the (311) facet of NiFe_2_O_4_, as the results of SAED in Figure S12c.


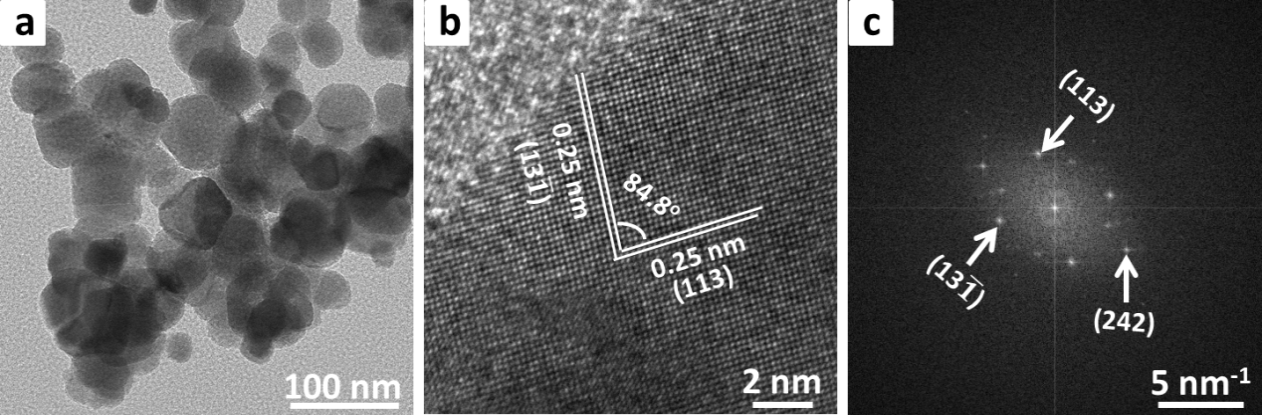


**Figure S12.** The TEM and SAED images of NiFeO.

The temperature for fabricating *d-*NiFe^HR^O/IF, during the solvothermal treatment is optimized. The intermediates were solvothermal-treated at 110 to 190 ^o^C, respectively. As a result, the sample solvothermal-treated at 150 ^o^C exhibits the best activity with an overpotential of 310 mV to reach the current density of 200 mA cm^-2^.


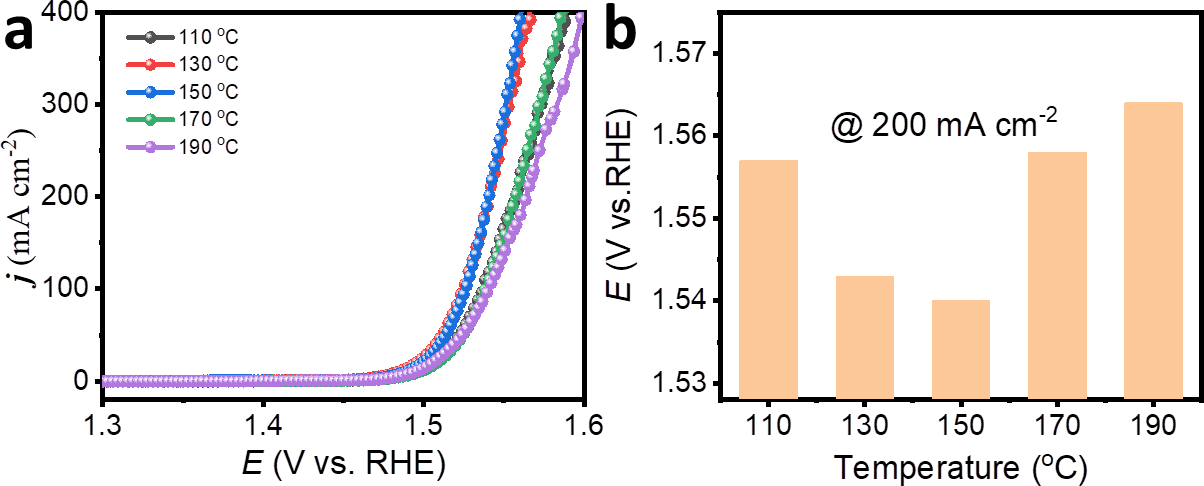


**Figure S13.** (a) The polarization curves of OER on samples solvothermal-treated at different temperatures. (b) The relationships between potentials at 200 mA cm^-2^ with the treated temperatures.

The time for fabricating *d-*NiFe^HR^O/IF , during the solvothermal treatment is optimized. The intermediates were solvothermal-treated for 18 to 42 h, respectively. As a result, the sample treated for 30 h exhibits the best activity with an overpotential of 310 mV to attain the current density of 200 mA cm^-2^.


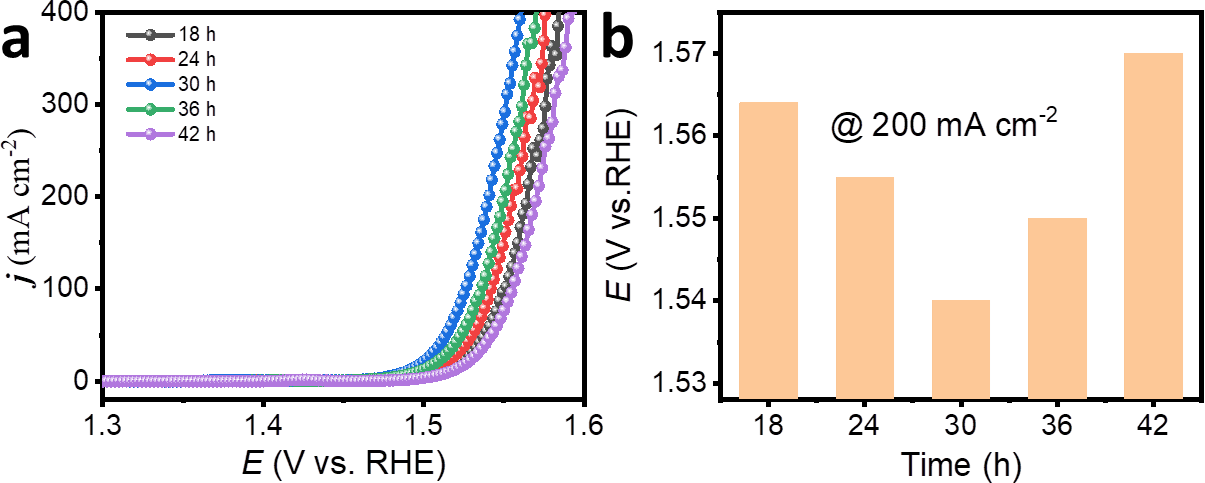


**Figure S14.** (a) The polarization curves of OER on samples solvothermal-treated for different times. (b) The relationships between potentials at 200 mA cm^-2^ with the treated time.

It is well known that specific surface areas (ECSA) of electrocatalysts played an important role in the electrochemical reaction process. Thus, ECSA is considered an important parameter for evaluating performances of electrocatalysts and clarifying the origin of catalytic activity. Generally, ECSA is estimated through electrochemical double layer capacitance (C_dl_) of electrocatalysts from CV curves in a non-Faradaic region with different scan rates:^[S12]^

C_dl_=$\frac{\boldsymbol{d}(\Delta\boldsymbol{j})}{\mathbf{2}\boldsymbol{dv}}$

In this formula, *Δj* represents the capacitive current density in selected potential on CV curves and $v$ is corresponding to scan rate. In a word, the slope of *Δj* against different scan rates can be used as parameter for evaluating its ECSA.^[S13]^ Relationships between *Δj* against different scan rates are obtained from corresponding CV cures on various electrocatalysts in 1.0 M KOH Figure S15. As shown in Figure S16, *d-*NiFe^HR^O/IF shows a largest ECSA of 11.39 mF cm^-2^. The larger ECSA of *d-*NiFe^HR^O/IF was attributed to the constructed geometrical defects as well as the optimized Fe-Ni atomic ratio by supplementation with Fe cations that increased the number of active sites.


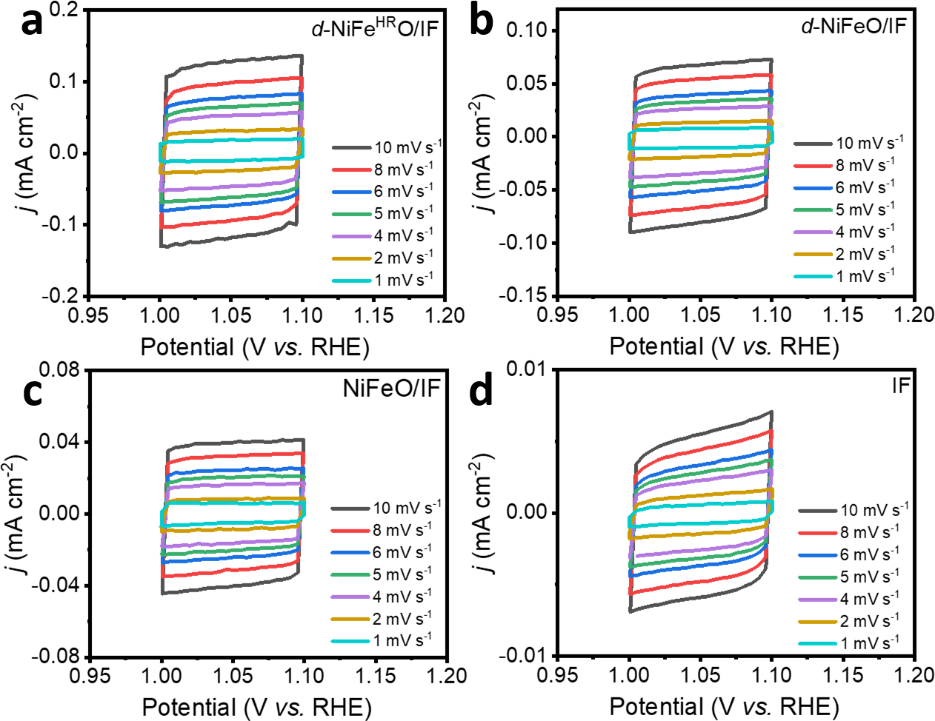


**Figure S15.** CV curves of (a) *d-*NiFe^HR^O/IF, (b) *d-*NiFeO/IF, (c) NiFeO/IF and (d) IF electrocatalysts in 1.0 M KOH from 1.0 to 1.1 V *vs*. RHE with scan rates from 1 to 10 mV s^-1^.


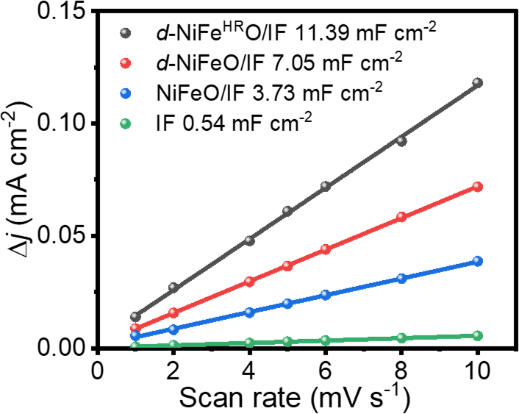


**Figure S16.** Relationships between capacitive current densities (*Δj*) measured on various electrocatalysts against different scan rates in 1.0 M KOH.

Nyquist plots of *d-*NiFe^HR^O/IF, *d-*NiFeO/IF, and NiFeO/IF electrocatalysts were performed to study kinetics of OER process. Experimental data determined from 1.45-1.55 V *vs*. RHE on various electrocatalysts in 1.0 M KOH were fitted by a two time constant model which is always described the mechanism of OER on porous electrodes (Figure S17 and 18). In general, charge transfer resistance (R_ct_) in two time constant model is used as an important parameter for activity of electrocatalysts and found to be overpotential dependent.^[S14]^ *d-*NiFe^HR^O/IF electrocatalysts exhibit lowest R_ct_ than that of *d-*NiFeO/IF and NiFeO/IF, which indicates minimum reaction resistance.


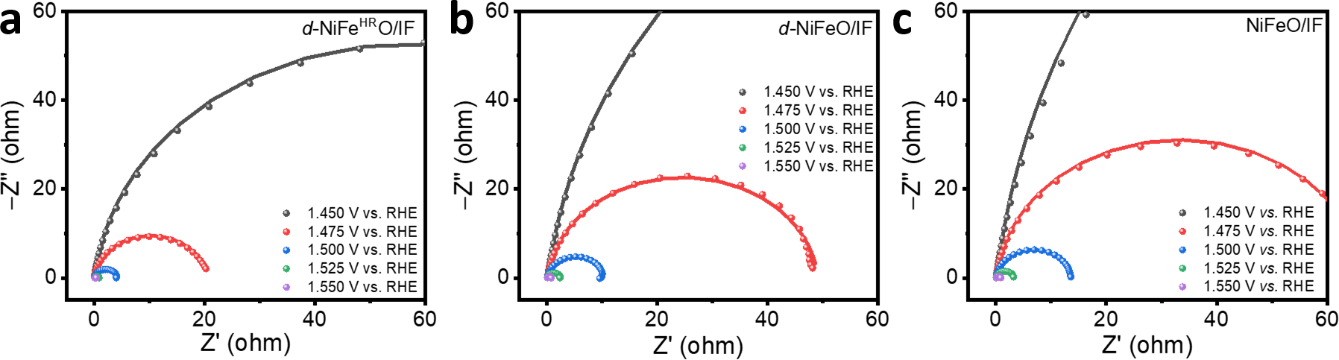


**Figure S17.** Nyquist plots of (a) *d-*NiFe^HR^O/IF, (b) *d-*NiFeO/IF, and (c) NiFeO/IF electrocatalysts for OER at various overpotentials in 1 .0 M KOH.


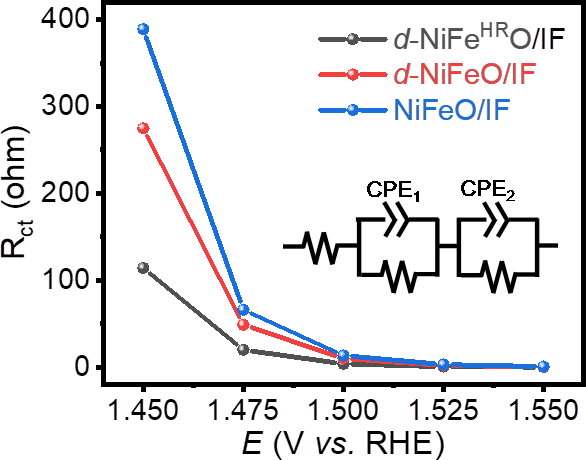


**Figure S18.** Relationships between resulting R_ct_ with corresponding overpotentials on various electrocatalysts in 1.0 M KOH

The XRD and SEM characterizations have been performed to investigate the structural stability of *d-*NiFe^HR^O/IF after the stability test (Figure S19 and 20). There is almost no change in XRD patterns and morphology of *d-*NiFe^HR^O/IF before and after the stability test. Meanwhile, surface reconstruction can be not observed. It indicates robust structural stability of *d-*NiFe^HR^O/IF in the OER process


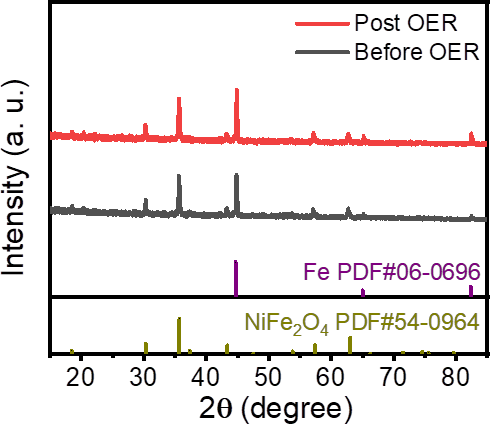


**Figure S19.** XRD patterns of *d-*NiFe^HR^O/IF before and after the stability test.


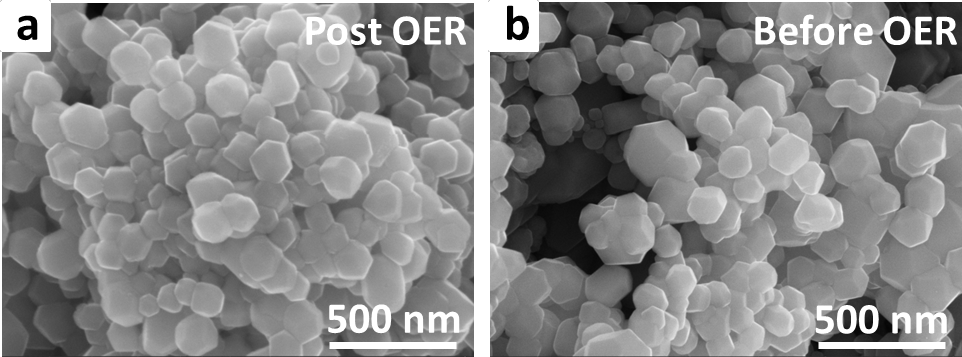


**Figure S20.** SEM images of *d-*NiFe^HR^O/IF before and after the stability test.

Electrochemical *in situ* FTIR spectroscopy was performed to reveal the adsorbed intermediates of the OER on NiFeO. As shown in Figure S21, when increasing the potential to approach the OER region (≥1.70 V *vs*. RHE), the *in situ* FTIR spectra of NiFeO show three characteristic absorption peaks at 1240, 1631, and 3402 cm^-1^, corresponding to the adsorbed intermediates of OOH*, O*, and OH*, respectively.


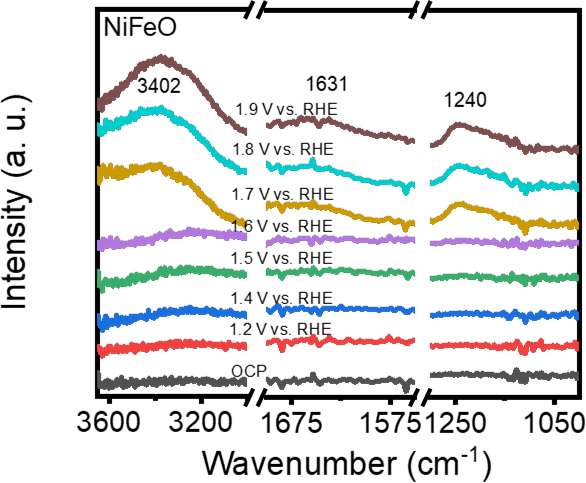


**Figure S21.** The *in situ* FTIR spectra of NiFeO electrocatalyst in 1 M KOH.


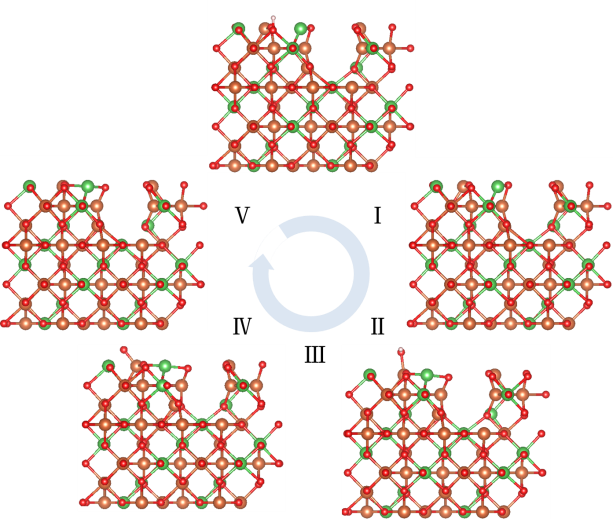


**Figure S22.** Optimized structures of key intermediates on *d-*NiFe^HR^O during the LOM pathway.


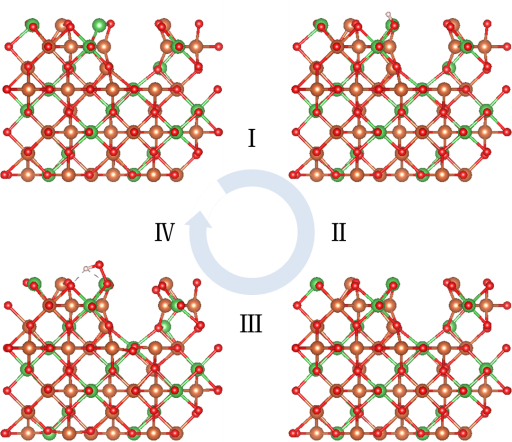


**Figure S23.** Optimized structures of key intermediates on *d-*NiFe^HR^O during the AEM pathway.


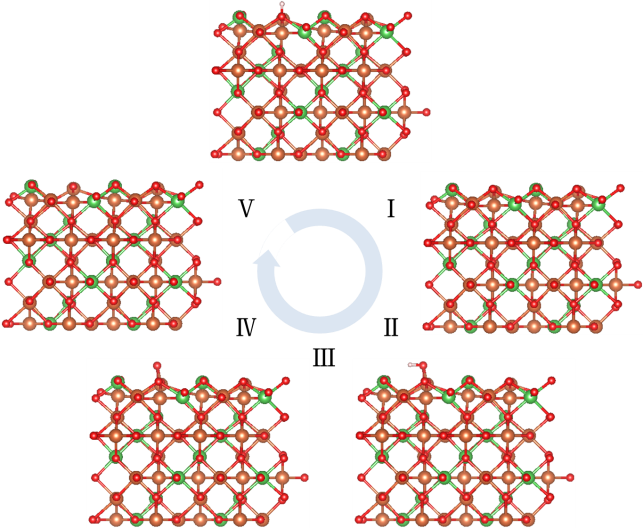


**Figure S24.** Optimized structures of key intermediates on NiFeO during the LOM pathway.


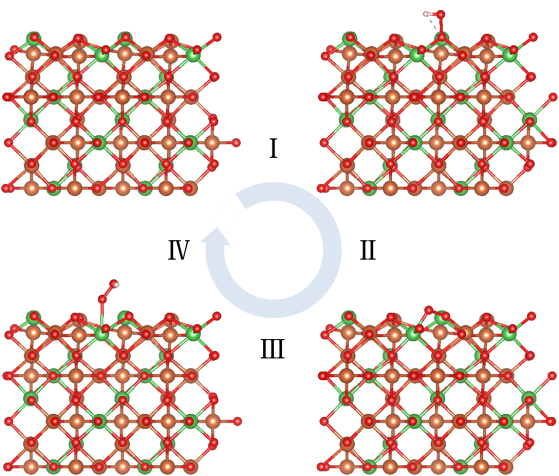


**Figure S25.** Optimized structures of key intermediates on NiFeO during the AEM pathway.

To clarify the role of surface reconstruction in triggering the LOM mechanism, the DFT simulations were performed to study the catalytic mechanism. A computational model of *d*-NiFe^HR^O with NiFeO_x_H_y_ formed on the surface was established to represent surface-reconstructed *d*-NiFe^HR^O (denoted as SR-*d*-NiFe^HR^O). As shown in Figure S26a, the first electrochemical deprotonation step in the LOM pathway is proven to be the PDS for both SR-*d*-NiFe^HR^O, which presents an energy barrier of 1.06 eV, even lower than that of *d*-NiFe^HR^O (1.38 eV). This indicates that the surface reconstruction can further enhance the OER activity on *d*-NiFe^HR^O. Furthermore, the difference between the Fe-*d* and O-*p* band centers (Δ_Fe/O_) is obtained to be 0.47 eV, which is very close to that of *d*-NiFe^HR^O (0.45 eV), suggesting that the covalency of the Fe-O bond has not changed significantly (Figure S26b).


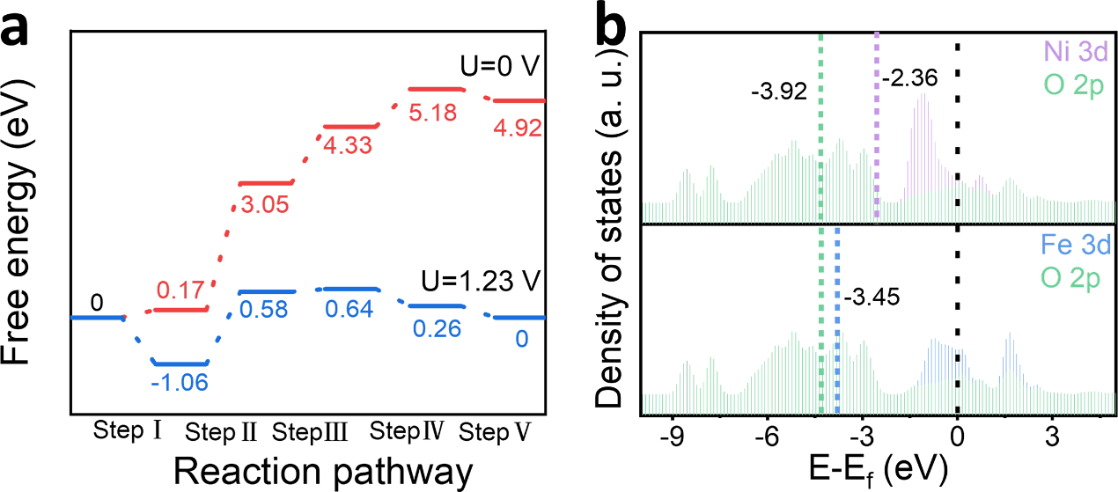


**Figure S26**. (a) OER Gibbs free energy diagrams based on SR-*d*-NiFe^HR^O through the LOM pathway. (b) PDOS of SR-*d*-NiFe^HR^O.


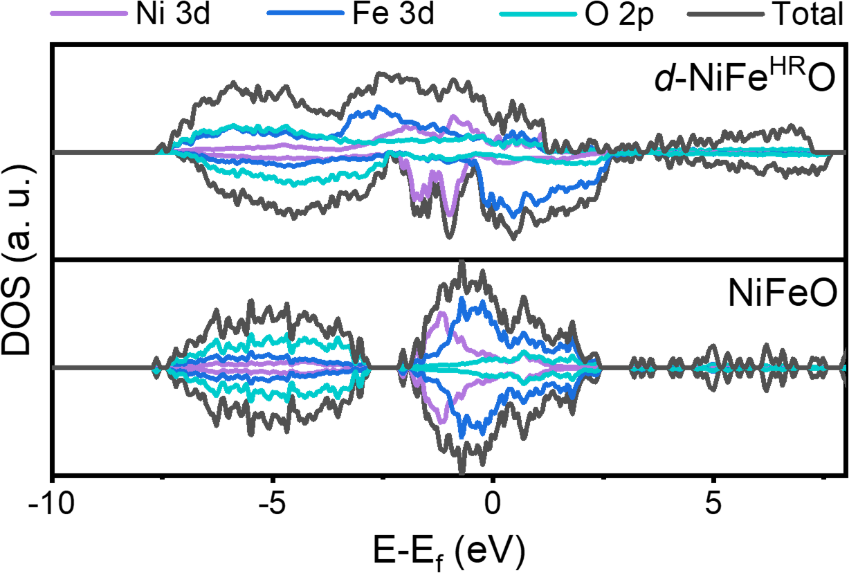


**Figure S27.** PDOS curves of NiFeO and *d*-NiFe^HR^O.

**3. Additional Tables**

**Table S1.** Contents and cationic ratios of Fe and Ni in *d*-NiFe^HR^O, *d*-NiFeO, and NiFeO detected by ICP-AES characterization.

|  | Fe | | Ni | |
| --- | --- | --- | --- | --- |
|  | mg | at% | mg | at% |
| *d*-NiFe^HR^O | 14.18 | 76.02 | 4.70 | 23.98 |
| *d*-NiFeO | 9.99 | 75.75 | 3.36 | 24.25 |
| NiFeO | 12.10 | 75.04 | 4.23 | 24.96 |

**Table S2.** Fitting parameters in the Fe K-edge EXAFS for *d*-NiFe^HR^O, *d*-NiFeO, and NiFeO.

| Materials | ΔE_0_ (eV) | S_0_^2^ | Shell | r (Å) | CN | σ^2^ (10^-3^ Å^2^ ) |
| --- | --- | --- | --- | --- | --- | --- |
| *d*-NiFe^HR^O | 3.491 | 0.800 | Fe_td_-O | 1.953 ± 0.017 | 3.988 | 6.960 |
|  |  |  | Fe_oct_-O | 2.085 ± 0.073 | 5.378 | 9.990 |
|  |  |  | Fe_oct_-TM_oct_ | 3.020 ± 0.072 | 5.342 | 10.070 |
|  |  |  | Fe_td_-TM_oct_ | 3.495 ± 0.039 | 8.125 | 17.84 |
|  |  |  | Fe_td_-TM_td_ | 3.638 ± 0.028 | 3.277 | -0.417 |
| *d*-NiFeO | 4.303 | 0.800 | Fe_td_-O | 1.954 ± 0.017 | 4.017 | 6.260 |
|  |  |  | Fe_oct_-O | 2.095 ± 0.084 | 5.038 | 8.190 |
|  |  |  | Fe_oct_-TM_oct_ | 3.031 ± 0.084 | 5.022 | 15.253 |
|  |  |  | Fe_td_-TM_oct_ | 3.503 ± 0.047 | 7.819 | 2.524 |
|  |  |  | Fe_td_-TM_td_ | 3.647 ± 0.037 | 2.102 | -1.012 |
| NiFeO | 3.861 | 0.800 | Fe_td_-O | 1.952 ± 0.016 | 3.988 | 6.420 |
|  |  |  | Fe_oct_-O | 2.087 ± 0.076 | 5.835 | 9.720 |
|  |  |  | Fe_oct_-TM_oct_ | 3.021 ± 0.073 | 5.768 | 10.07 |
|  |  |  | Fe_td_-TM_oct_ | 3.498 ± 0.042 | 8.321 | 1.769 |
|  |  |  | Fe_td_-TM_td_ | 3.642 ± 0.032 | 3.628 | 0.264 |

**Table S3** Comparisons of OER activities on *d*-NiFe^HR^O/IF and recent reported state-of-the-art Ni-Fe electrocatalysts.

| Catalysis | η_10_ (mV) | Tafel slope (mV dec^-1^) | Electrolyte | Ref. |
| --- | --- | --- | --- | --- |
| Ti-NFO | 230 | 74 | 1 M KOH | *ACS Catal.* 2024, **14,** 4453. |
| S-NFO NS | 261 | 84.2 | 1 M KOH | *ACS Energy Lett.* 2023, **8** 3504. |
| NiFe_2_O_4_/Ni_2_P | 230 | 56.3 | 1 M KOH | *Int. J. Hydrogen Energy* 2024, **51,** 770. |
| S- NiFe_2_O_4_/NF | 267 | 36.7 | 1 M KOH | *Nano Energy* 2017, **40,** 264. |
| NiFe_2_O_4_-H_2_ | 389 | 64 | 1 M KOH | *ChemNanoMat* 2019, **5** 1296. |
| NiS/NiFe_2_O_4_ | 230 | 88 | 1 M KOH | *Adv Mater* 2020, **34** 2110172. |
| MoS_2_/rFe-NiCo_2_O_4_ | 270 | 39 | 1 M NaOH | *J. Am. Chem. Soc.* 2020, **142** 50. |
| Ni-Fe LDH Nanosheets | 280 | 49.4 | 1 M KOH | *Angew. Chem. Int. Ed.* 2018, **57** 172. |
| Fe_2_O_3_/Fe_0.64_Ni_0.36_@C-800 | 274 | 82.98 | 1 M KOH | *Small* 2023, **19,** 2208276. |
| NiFe LDH@CuxO | 270 | 67 | 1 M KOH | *Int. J. Hydrogen Energy* 2023, **48** 4719. |
| Ni-Fe LDH DSNCs | 246 | 117 | 1 M KOH | *Adv. Mater.* 2020, **32** 1906432. |
| NiFe@PCN | 310 | 38 | 1 M KOH | *J. Mater. Chem. A* 2019, **7** 14001. |
| Fe_0.2_Ni_0.8_/NC-600-a | 290 | 76 | 1 M KOH | *Chem. Eng. J.* 2020, **394** 124977. |
| NiFe@CN-G | 320 | 41 | 1 M KOH | *ChemElectroChem* 2018, **5** 732. |
| NiFe@NCNFs | 294 | 52 | 1 M KOH | *ACS Appl. Mater. Interfaces* 2020, **12** 31503. |
| *d*-NiFe^HR^O | 258 | 34.6 | 1 M KOH | This Work |

References

[S1] B. Ravel, M. Newville, *J. Synchrotron Radiat.* **2005**, 12, 537.

[S2] G. Kresse, D. Joubert, *Phys. Rev. B* **1999**, 59, 1758.

[S3] J. P. Perdew, K. Burke, M. Ernzerhof, *Phys. Rev. Lett.* **1996**, 77, 3865.

[S4] S. Grimme, J. Antony, S. Ehrlich, H. Krieg, *J. Chem. Phys.* **2010**, 132, 154104.

[S5] a) Y. Wang, X. Ge, Q. Lu, W. Bai, C. Ye, Z. Shao, Y. Bu, *Nat. Commun.* **2023**, 14, 6968; b)H. You, D. Wu, D. Si, M. Cao, F. Sun, H. Zhang, H. Wang, T. F. Liu, R. Cao, *J. Am. Chem. Soc.* **2022**, 144, 9254.

[S6] a) L. Yi, S. Xiao, Y. Wei, D. Li, R. Wang, S. Guo, W. Hu, *Chem. Eng. J.* **2023**, 469, 144015; b) X. Li, H. Huang, B. Chen, *Ind. Eng. Chem. Res.* **2023**, 63, 296.

[S7] W. Zhang, Y. Shen, J. Zhang, H. Bi, S. Zhao, P. Zhou, C. Han, D. Wei, N. Cheng, *Appl. Surf. Sci.* **2019**, 470, 581.

[S8] a) B. Talluri, K. Yoo, J. Kim, *J. Environ. Chem. Eng.* **2022**, 10, 106932; b) D. Liu, C. Huyan, Y. Zhao, T. X. Liu, D. Wang, J. Sun, S. Dai, F. Chen, B. B. Xu, *Adv. Mater. Interfaces* **2022**, 9, 2200276.

[S9] a) B. Zhang, Z. Wu, W. Shao, Y. Gao, W. Wang, T. Ma, L. Ma, S. Li, C. Cheng, C. Zhao, *Angew Chem. Int. Ed.* **2022**, 61, e202115331; b) M. Yao, H. Hu, N. Wang, W. Hu, S. Komarneni, *J. Colloid Interface Sci.* **2020**, 561, 576; c) J. F. Marco, J. R. Gancedo, J. Ortiz, J. L. Gautier, *Appl. Surf. Sci.* **2004**, 227, 175.

[S10] a) J. Song, J. Zhang, A. Zada, Y. Ma, K. Qi, *Ceram. Int.* **2023**, 49, 12327; b) P. Chen, C. Cao, C. Ding, Z. Yin, S. Qi, J. Guo, M. Zhang, Z. Sun, *J. Power Sources* **2022**, 521, 230920; c) F. Li, M. L. Li, H. F. Wang, X. X. Wang, L. J. Zheng, D. H. Guan, L. M. Chang, J. J. Xu, Y. Wang, *Adv. Mater.* **2022**, 34, 2107826.

[S11] a) B. Tong, G. Meng, Z. Deng, M. Horprathum, A. Klamchuen, X. Fang, *Chem. Commun.* **2019**, 55, 11691; b) L. He, H. Kang, G. Hou, X. Qiao, X. Jia, W. Qin, X. Wu, *Chem. Eng. J.* **2023**, 460, 141675; c) Y. Li, F.-M. Li, X.-Y. Meng, S.-N. Li, J.-H. Zeng, Y. Chen, *ACS Catal.* **2018**, 8, 1913.

[S12] A. Sivanantham, P. Ganesan, S. Shanmugam, *Adv. Funct. Mater.* **2016**, 26, 4661.

[S13] J. Zhang, J. Qian, J. Ran, P. Xi, L. Yang, D. Gao, *ACS Catal.* **2020**, 10, 12376.

[S14] Y. J. Wu, J. Yang, T. X. Tu, W. Q. Li, P. F. Zhang, Y. Zhou, J. F. Li, J. T. Li, S. G. Sun, *Angew Chem. Int. Ed.* **2021**, 60, 26829.
